# Comparative effectiveness of rhythmic grip training of various intensities on cognitive function in young adults: a fNIRS study

**DOI:** 10.3389/fnins.2025.1722639

**Published:** 2025-12-01

**Authors:** Huming Zhu, Yanbai Han, Mengzhao Wang, Zhuoyue Cheng, Mingrui Du, Huilin Jiang, Hongli Wang

**Affiliations:** College of Physical Education and Health, Guangxi Normal University, Guilin, Guangxi, China

**Keywords:** rhythmic handgrip, exercise intensity, cognitive performance, fNIRS, neural efficiency

## Abstract

**Introduction:**

Resistance training has a significant influence on cognitive function. Rhythmic handgrip (RHG) exercise, a practical form of small-muscle group training, may improve cognitive performance. However, the effects of resistance training at various intensities on prefrontal cortex (PFC) activation and cognitive outcomes remain unclear.

**Methods:**

This study recruited 24 healthy male participants (mean age: 22.39 ± 1.31 years). Participants completed experiments at three intensity levels [30%, 50%, and 70% Maximum Voluntary Contraction (MVC)] in a randomized order, with one-week intervals between sessions. During dynamic handgrip resistance training, participants performed rhythmic fist contractions at the target intensity, maintaining a frequency of 1 Hz for 30 s, followed by 30 s of rest. Each training set consisted of four contraction-rest cycles, and participants completed three sets in total, separated by 3-min rest periods. Cognitive task testing including the Stroop task, 2-Back task, and More-Odd Shifting task, was administered before and after each intervention. Cognitive performance was evaluated using reaction time and accuracy measures. Simultaneously, functional near-infrared spectroscopy was employed to measure changes in oxygenated hemoglobin (HbO_2_) levels in the prefrontal cortex during task execution.

**Results:**

The moderate-intensity (50% MVC) rhythmic handgrip task produced the greatest improvements in cognitive performance. Behaviorally, participants showed faster reaction times and higher accuracy than in low- (30% MVC) or high-intensity (70% MVC) conditions. Functional near-infrared spectroscopy results showed that moderate-intensity rhythmic handgrip enhanced the efficiency of prefrontal modulation, particularly in the dorsolateral prefrontal cortex and inferior frontal gyrus. The 50% MVC condition reduced oxygenated hemoglobin levels, indicating optimized neural efficiency and more effective cortical resource allocation during task performance.

**Conclusion:**

Moderate-intensity (50% MVC) RHG training significantly enhances executive function, working memory, and cognitive flexibility, while demonstrating more efficient neural activity. As a practical form of small-muscle-group training, moderate-intensity RHG resistance offers a promising early-intervention strategy for delaying cognitive decline and reducing dementia risk.

**Clinical trial registration:**

https://www.chictr.org.cn, identifier ChiCTR2400083689.

## 1 Introduction

Maintaining cognitive health across the lifespan has become a major public health goal. Among the various age-related cognitive disorders, dementia represents one of the most serious global challenges. Dementia is a progressive neurodegenerative disorder characterized by a gradual decline in cognitive function, with the number of affected individuals projected to increase from 57.4 million in 2019 to 152.8 million by 2050. This rising prevalence is expected to strain healthcare systems substantially and pose significant challenges for families and societies worldwide (Banerjee et al., 2006; Nichols et al., 2022). Neuropathological changes associated with dementia often begin years before the onset of clinical symptoms. Currently, no curative treatments are available, and pharmacological interventions offer only modest symptomatic relief (Cummings et al., 2016). Consequently, prevention and early intervention have become central priorities in dementia research (Demurtas et al., 2020; Dieckelmann et al., 2023). The cognitive reserve theory posits that accumulating cognitive reserve earlier in life is critical for reducing the risk of dementia (Stern, 2009; Wang et al., 2020; Liu et al., 2024). Education and physical activity are key modifiable factors contributing to cognitive reserve (Association, 2024). Education enhances neural plasticity and supports sustained cognitive performance across the lifespan (Nelson et al., 2021). Physical activity promotes lifelong cognitive health by improving cardiovascular function, enhancing metabolic regulation, and increasing neurotrophic support (de Rooij, 2022; Weuve et al., 2013; Lautenschlager et al., 2008; Erickson et al., 2011).

The impact of exercise on cognitive function depends on multiple elements, including volume, modality, frequency, duration, and intensity. Aerobic exercise enhances cerebral blood flow and supports neuroplasticity. However, its effectiveness typically requires high training volume and long-term adherence, which may limit its feasibility and accessibility for vulnerable populations (Huang et al., 2022; Zhang et al., 2013; Hillman et al., 2008; Brown et al., 2012; Colcombe and Kramer, 2003; Erickson et al., 2011; Northey et al., 2018; Bherer et al., 2013). Compared with other forms of exercise, resistance training allows precise regulation of intensity and cardiovascular effort, enhances neural activation, and ensures safety and broad applicability. Consequently, it has become a central topic in cognitive intervention research (Stillman et al., 2016; Liu-Ambrose et al., 2012; Cassilhas et al., 2007). Among all exercise variables, exercise intensity exerts a particularly pronounced effect on cognitive outcomes, and this influence is independent of total exercise volume. Although numerous studies have examined the cognitive benefits of resistance training, the specific role of exercise intensity remains uncertain. Differences in study design, control of exercise volume, and target populations have led to mixed findings, making it challenging to determine how intensity, per se, affects cognitive outcomes (Baker et al., 2012; Angevaren et al., 2007; Mann et al., 2013; Batman et al., 2024; Brush et al., 2016).

This study adopted Rhythmic handgrip (RHG) exercise as a model to precisely examine the effect of resistance intensity under controlled conditions. Compared with whole-body resistance exercise, localized small-muscle training such as resistance band exercises or handgrip device use has been demonstrated to be equally effective while offering greater practicality (Sanchez-Lastra et al., 2013; Zhu et al., 2022). Compared with whole-body exercise, localized RHG training offers a simple, well-controlled, and quantifiable model to isolate intensity-dependent effects on cerebral and cognitive functions, while minimizing confounding influences from systemic fatigue or whole-body metabolic stress. However, its influence on cerebral hemodynamics and executive performance has not been extensively characterized, leaving a critical gap in understanding how localized rhythmic contractions modulate brain function compared. The rhythmic contraction–relaxation pattern of RHG produces pulsatile oscillations in peripheral and cerebral blood flow, which are proposed to enhance cerebrovascular autoregulation and oxygen delivery to the prefrontal cortex (PFC). Previous research has demonstrated that submaximal-intensity RHG training enhances oxygen delivery to the prefrontal cortex while reducing subjective fatigue (Dallaway et al., 2023). In older adults, vascular responses induced by handgrip training are associated with a reduced white matter hyperintensity burden, indicating potential neuroprotective benefits (Pearson et al., 2022). Therefore, RHG can serve as a practical model for investigating how resistance training intensity modulates neural efficiency and executive function. Functional near-infrared spectroscopy (fNIRS) is a reliable tool for real-time monitoring of cerebral oxygenation. This technique aids in elucidating the mechanisms by which rhythmic movements of small muscle groups enhance cognitive function (Noah et al., 2015; Arenth et al., 2007).

Based on these characteristics, we proposed two hypotheses. First, moderate-intensity RHG at 50% Maximum Voluntary Contraction (MVC) leads to the most significant improvement in executive functions, including inhibitory control, cognitive flexibility, and working memory, compared with low-intensity (30% MVC) and high-intensity (70% MVC) conditions. Second, these behavioral improvements would be accompanied by more efficient patterns of prefrontal activation. This efficiency would be reflected by focused and reduced HbO_2_ responses within task-related cortical regions as measured by fNIRS.

Therefore, this study examines how RHG exercise at different intensities influences prefrontal activation and executive performance under conditions where the total work performed increases with intensity. By integrating behavioral and neurophysiological measures, it aims to clarify the relationship between exercise intensity, neural efficiency, and cognitive function.

## 2 Materials and methods

### 2.1 Study design

This self-controlled repeated-measures study was conducted from September 2024 to May 2025 and added to the China Clinical Trial Registry on April 30, 2024. All results were presented thoroughly in accordance with the CONSORT 2010 principles, and the study procedure was predetermined and closely followed.

### 2.2 Participants

A total of 24 young male participants were recruited and screened per the established inclusion and exclusion criteria; no withdrawals occurred throughout the experimental procedure. At baseline, the participants’ mean age was 22.39 ± 1.31 years, and their mean BMI was 21.88 ± 2.64 kg/m^2^. Their maximal voluntary contraction of the right hand was 34.48 ± 11.16 kg. All participants were right-handed, had normal or corrected-to-normal vision, and had no neurological or psychiatric disorders history. They also met the following inclusion criteria: (1) they were between 20 and 25 years old, physically healthy, and had no history of hypertension, other severe diseases, brain injury, or neurosurgery; (2) they did not consume coffee, tea, or other beverages within 24 h prior to testing; (3) good mental health, with no history of drug or alcohol dependence; (4) normal or corrected-to-normal vision, without color blindness or color weakness;(5) all participants were healthy young adults without any history of neurological disorders or professional athletic training. None had prior experience with RHG or similar motor tasks, ensuring that the results reflected untrained individuals’ responses to the intervention.

All participants were explained the experiment’s goals and methods prior to its start, and written informed consent was obtained. Guangxi Normal University’s Ethics Committee accepted the study (Approval Number: 20231226001), and all procedures followed the most recent version of the Declaration of Helsinki’s rules and regulations.

### 2.3 Experimental procedures

This study investigated how RHG training at various intensities, while conditions where the total work performed increases with intensity. Affects PFC activity during subsequent executive function tasks. The fNIRS measured changes in HbO_2_ in the prefrontal area during three cognitive tasks: the 2-Back task, the Stroop task, and the More-Odd Shifting task. The study was structured into three phases: pre-intervention testing, the intervention itself, and post-intervention testing. Each participant was required to complete three experimental sessions.

The MVC test was performed on a separate day before the initial intervention session to avoid any fatigue from the MVC assessment influencing the first intervention. Measurements were obtained with a calibrated handgrip dynamometer under standardized posture and consistent environmental conditions. They were placed in a standardized posture: seated position with the shoulder at 0° abduction, elbow flexed at 90°, forearm in a neutral position, and wrist extended at 0–30° with slight ulnar deviation. Prior to the measurements, a brief warm-up and demonstration were conducted to ensure that the participants understood the exertion method. During the testing, participants gripped the handle with maximal effort for about 3–5 s, while receiving verbal encouragement to ensure maximum output. Each hand was tested 2–3 times, with the highest value recorded as the maximum grip strength index. Intervals of 30–60 s were maintained between tests to prevent fatigue. Data reliability and comparability were ensured through consistency in environment, posture, and measurement equipment. On that same day, all participants practiced the three cognitive paradigms (Stroop, 2-Back, and More–Odd Shifting) repeatedly until their performance stabilized. This ensured that baseline measurements accurately reflected task proficiency and were not influenced by initial learning effects.

During the pre-intervention testing phase, participants entered the laboratory and spent 15 min adapting to a quiet environment to minimize stress and external interference. The testing sessions were scheduled between 9:00 a.m. and 12:00 a.m. Participants then completed cognitive tasks, including the 2-Back, Stroop, and More-Odd Shifting tasks, presented randomly to control for sequence effects. A 19-channel fNIRS system was used to record changes in HbO_2_ levels in the prefrontal cortex. The investigators ensured proper contact with the scalp, continuously monitored signal quality, and removed noisy or invalid channels. Follow with a brief 2–3 min warm-up of the forearms and hands, familiarising yourself with the contraction rhythm using a metronome to ensure the target intensity remains consistent throughout each cycle.

During the intervention phase, each participant completed three distinct intensity levels of intervention training: low intensity (30% of MVC), moderate intensity (50% of MVC), and high intensity (70% of MVC). The sequence of these intensities was randomized, with 1 week’s interval between each session. During RHG training, participants calibrated their target intensity using an adjustable dynamometer, performing rhythmic contractions at 1 Hz for 30 s followed by 30 s of rest. Each training set comprised four contraction-rest cycles, with participants completing three sets separated by 3-min rest intervals. An 18-min dynamic handgrip training session. All participants completed the training using their dominant hand (right hand).

All participants underwent immediate post-testing following the intervention completion, performing identical cognitive tasks to the pre-test under consistent environmental conditions and equipment settings (Chang et al., 2017). Testing duration remained consistent with the pre-intervention phase. Following completion of all testing, hand relaxation exercises were performed (gentle shaking/relaxation, palm warming with three deep breaths, wrist circles, finger spreading-and-retraction, thumb-and-finger opposition).

**Figure 1:**
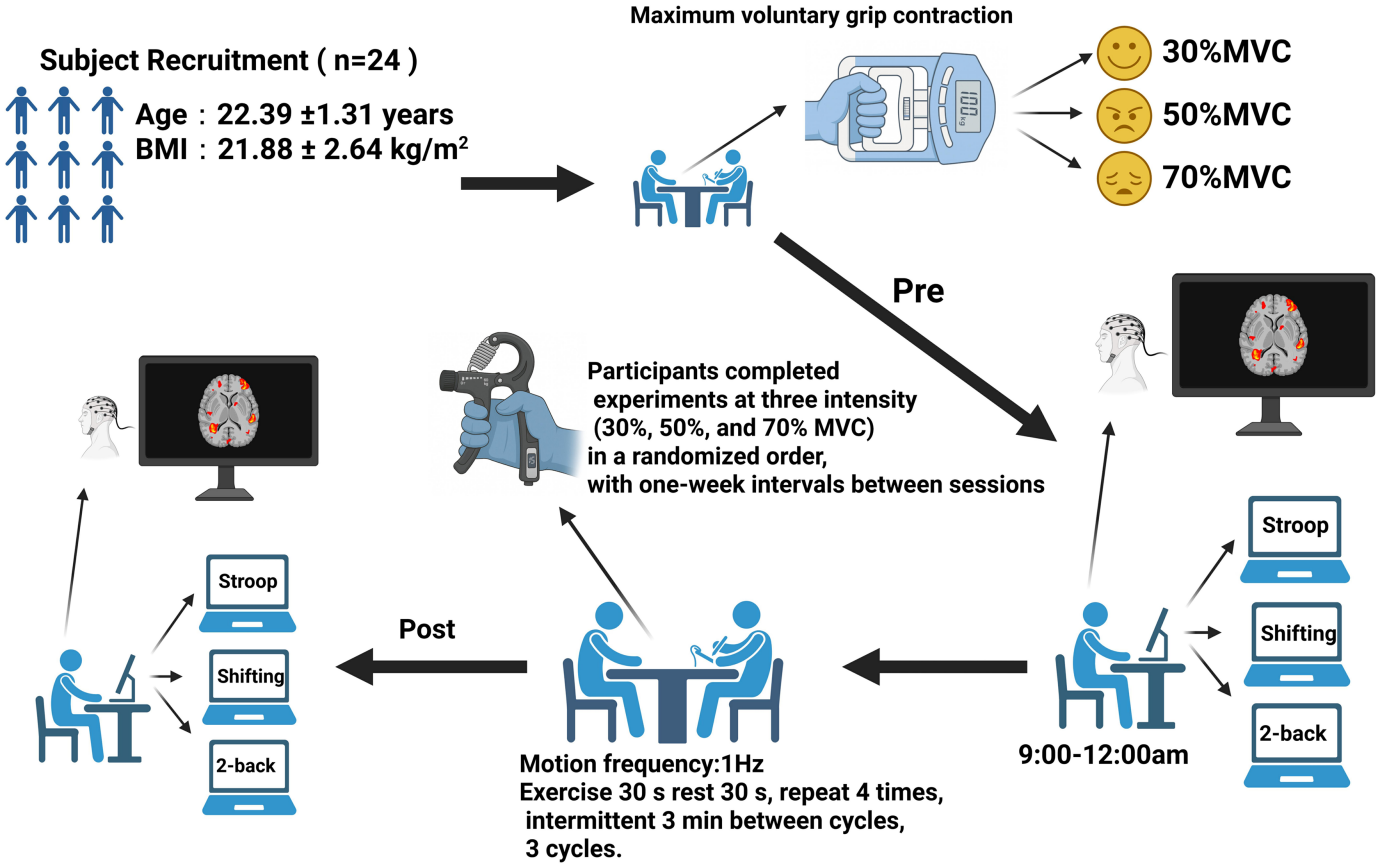
Experimental procedure chart (*n* = 24). Change in oxygenated hemoglobin concentration.

During each intervention session, once the fNIRS cap was positioned correctly and the signal quality verified, the optical probe remained in place throughout the entire experimental process, including both the pre- and post-intervention cognitive testing. The probe was not removed or adjusted between tests. This ensured consistent probe–scalp coupling and prevented measurement variability caused by reattachment or repositioning of the probe. The same cap size, optode configuration, and placement reference points (based on the international 10–20 system) were used across sessions for each participant (Figure 1).

### 2.4 Blinding and bias control

Different researchers were assigned specific roles during the study to minimize experimenter bias. One group of instructors supervised the intervention sessions, while another group of experimenters conducted the pre- and post-intervention cognitive testing and fNIRS data collection, remaining blind to group assignments. The data analysts were also blinded to group allocation during the statistical analysis, with datasets identified solely by ID numbers. Additionally, all participants followed standardized testing protocols and predefined analytical pipelines to limit subjective bias further.

### 2.5 fNIRS data acquisition

**Figure 2:**
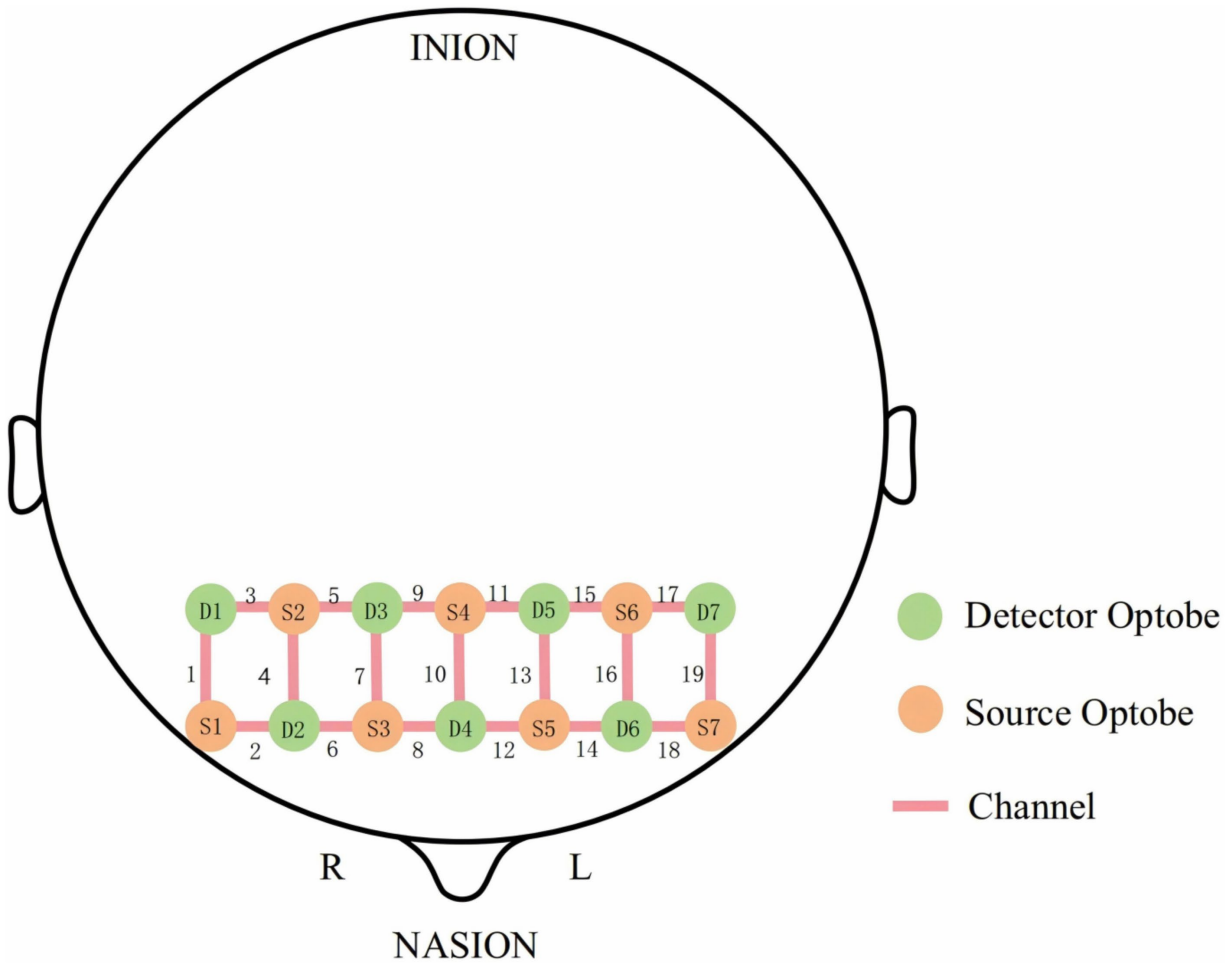
Placement of optode in 19 channels of the prefrontal lobe of the brain based on the international 10/20 system.

This study utilized a portable functional near-infrared brain imaging device (NirSmartII-3000C, Huichuang Medical Equipment Co., Ltd., Danyang, China) to collect fNIRS signals. The device featured dual near-infrared light sources operating at 730 nm and 850 nm wavelengths, with a sampling frequency of 11 Hz. The probe arrangement adhered to the international 10–20 system and included seven emitters and seven detectors. This configuration created 19 measurement channels focused on the prefrontal cortex, allowing for the monitoring of hemodynamic changes in this brain region (Figure 2).

The anatomical structure of the prefrontal cortex and the distribution of the 19 channels led to their categorization into six regions of interest (ROIs): the right inferior frontal gyrus (RIFG), left inferior frontal gyrus (LIFG), right dorsolateral prefrontal cortex (RDLPFC), left dorsolateral prefrontal cortex (LDLPFC), right frontopolar area (RFPA), and left frontopolar area (LFPA) (Rorden and Brett, 2000). This division was designed to facilitate specific signal extraction and analysis of the different functional subregions within the prefrontal cortex (Table 1).

**Table 1:** ROI corresponding to each channel.

Region of Interest (ROI)	Channels (CH)
Right inferior prefrontal gyrus (RIFG)	CH1, CH2
Left inferior prefrontal gyrus (LIFG)	CH18, CH19
Right dorsolateral prefrontal cortex (RDLPFC)	CH3, CH4, CH5, CH6, CH7
Left dorsolateral prefrontal cortex (LDLPFC)	CH13, CH14, CH15, CH16, CH17
Right frontopolar area (RFPA)	CH8, CH9
Left frontopolar area (LFPA)	CH11, CH12

### 2.6 Experimental design paradigms

Three well-established paradigms were employed to evaluate the core executive functions, specifically targeting inhibitory control, working memory, and cognitive flexibility. All tasks were administered using E-Prime software, comprising three blocks of 48 trials, resulting in 144 trials per task. Each trial commenced with a fixation point (“+”) displayed for 500 ms, followed by presenting a stimulus image for 1,000 ms. A rest period of 30 s was provided between blocks to mitigate fatigue. The specific designs of the tasks are detailed as follows. Each block lasted approximately 90 s, followed by a 30-s rest block to allow hemodynamic recovery. Between consecutive tasks, participants rested for 2 min before starting the next paradigm. The total duration of each cognitive task, including rest periods, was roughly 7–8 min (Parris et al., 2022; Zohdi et al., 2024; Dong et al., 2023).

#### 2.6.1 Inhibitory control—Stroop task

**Figure 3:**
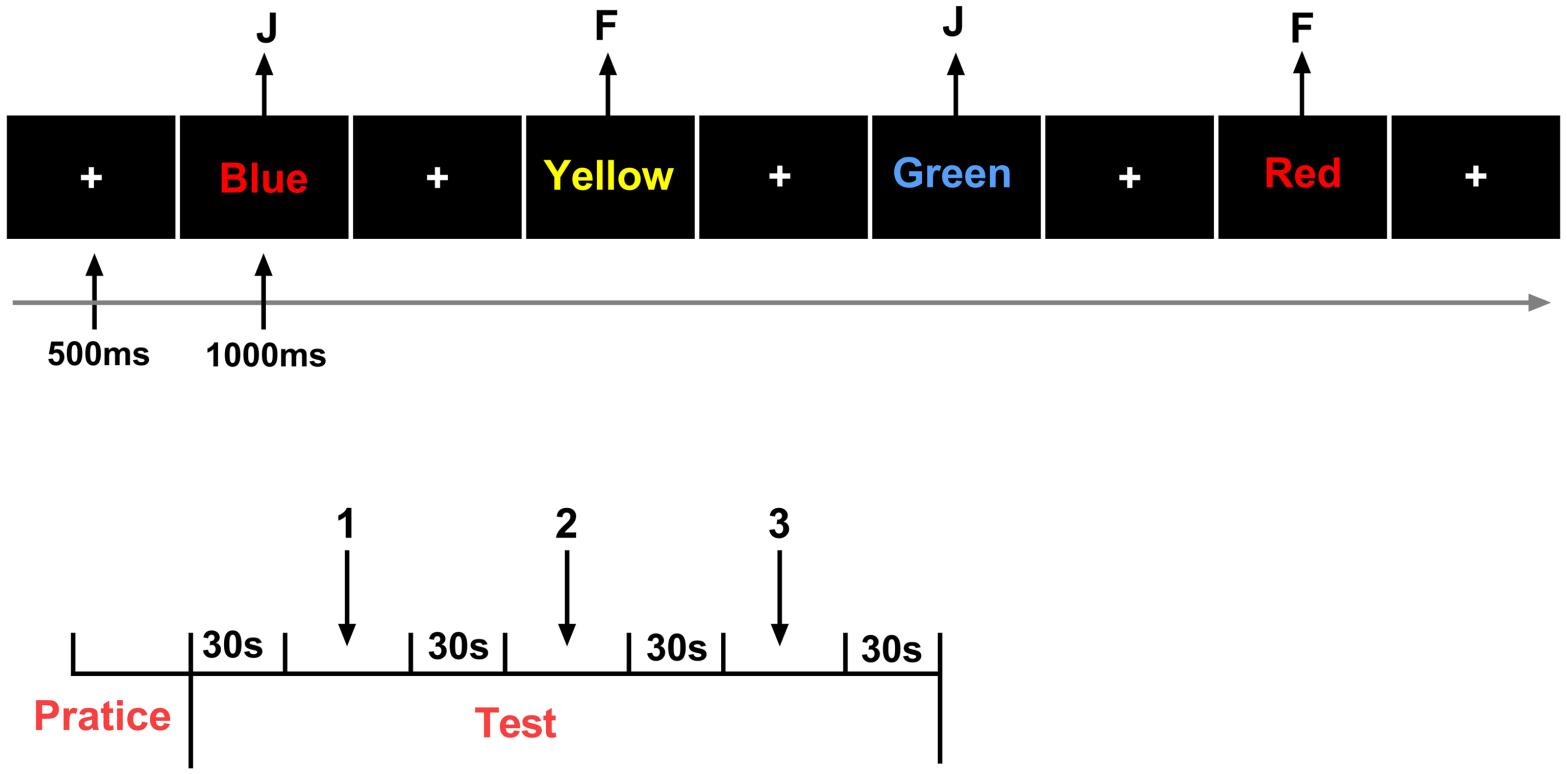
Schematic of the Stroop task procedure.

The Stroop task was used to assess inhibitory control. During each trial, a color word (e.g., RED, BLUE, GREEN) was displayed in either a congruent or incongruent ink color. Participants were required to identify the font color while ignoring the word meaning. Behavioral data were collected for both congruent and incongruent conditions, including reaction time (RT) and accuracy, to quantify inhibitory performance and Stroop interference effects (Figure 3).

#### 2.6.2 Cognitive flexibility—More Odd Shifting task

**Figure 4:**
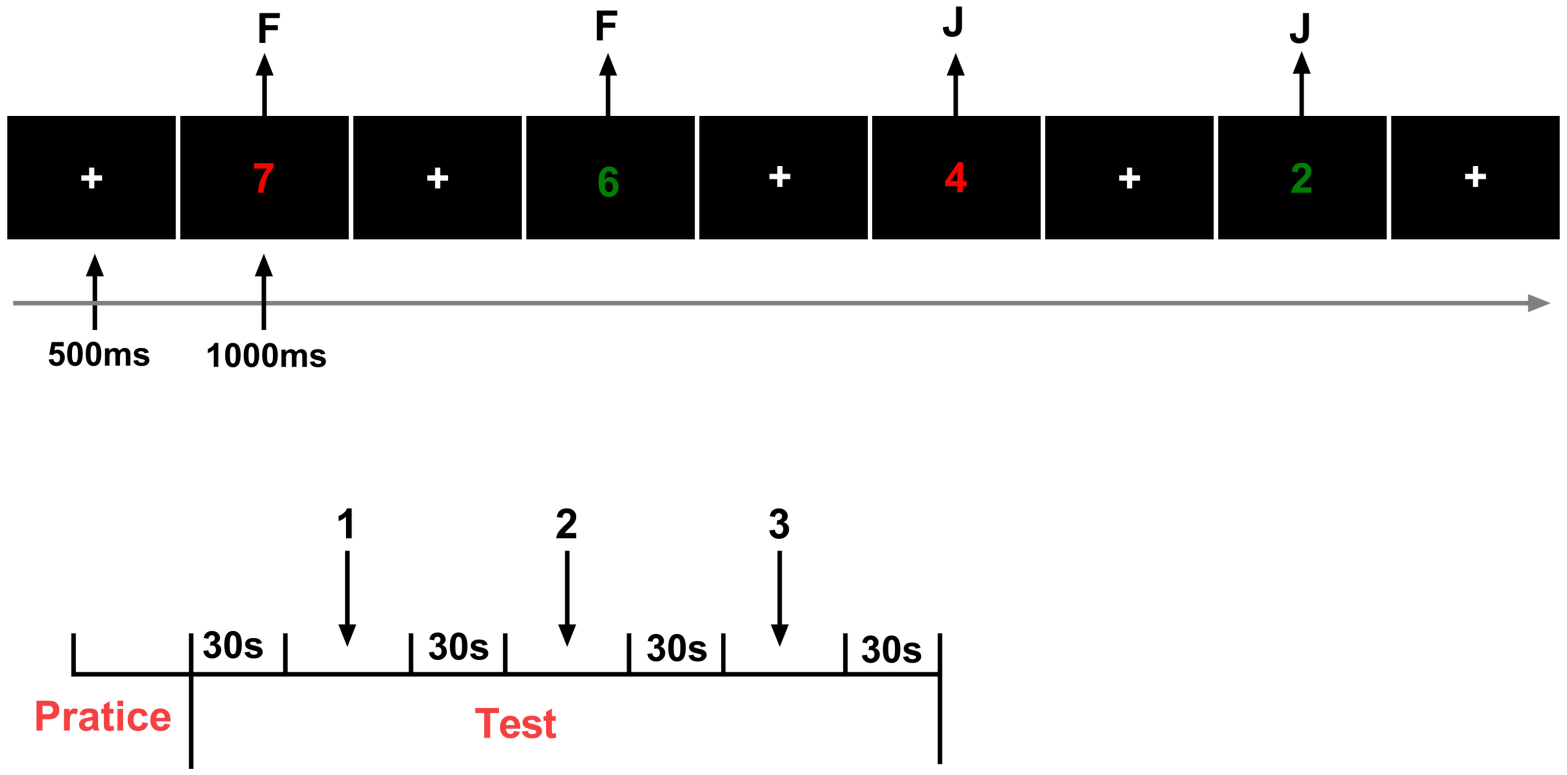
Schematic of the More-Odd Shifting task procedure.

The More Odd Shifting task was employed to assess cognitive flexibility. In this task, digits ranging from 1 to 9 (excluding 5) were presented in either green or red font color, with the color serving as a visual cue that indicated the applicable judgment rule. When the number appeared in red, participants were instructed to determine whether it was odd or even, whereas when the number appeared in red, they judged whether it was greater or less than five. Each sequence consisted of three consecutive trials that followed a fixed pattern: the first two trials required the same judgment rule, referred to as non switching trials, and the third trial required a change of rule, constituting a switching trial. This periodic presentation design systematically elicited rule switching and allowed for the evaluation of cognitive shifting ability. Behavioral performance was assessed by measuring RT and accuracy for both switching and non switching conditions, with the switch cost, calculated as the RT difference between switching and non switching trials, serving as an indicator of cognitive flexibility efficiency (Figure 4).

#### 2.6.3 Working memory—2-Back task

**Figure 5:**
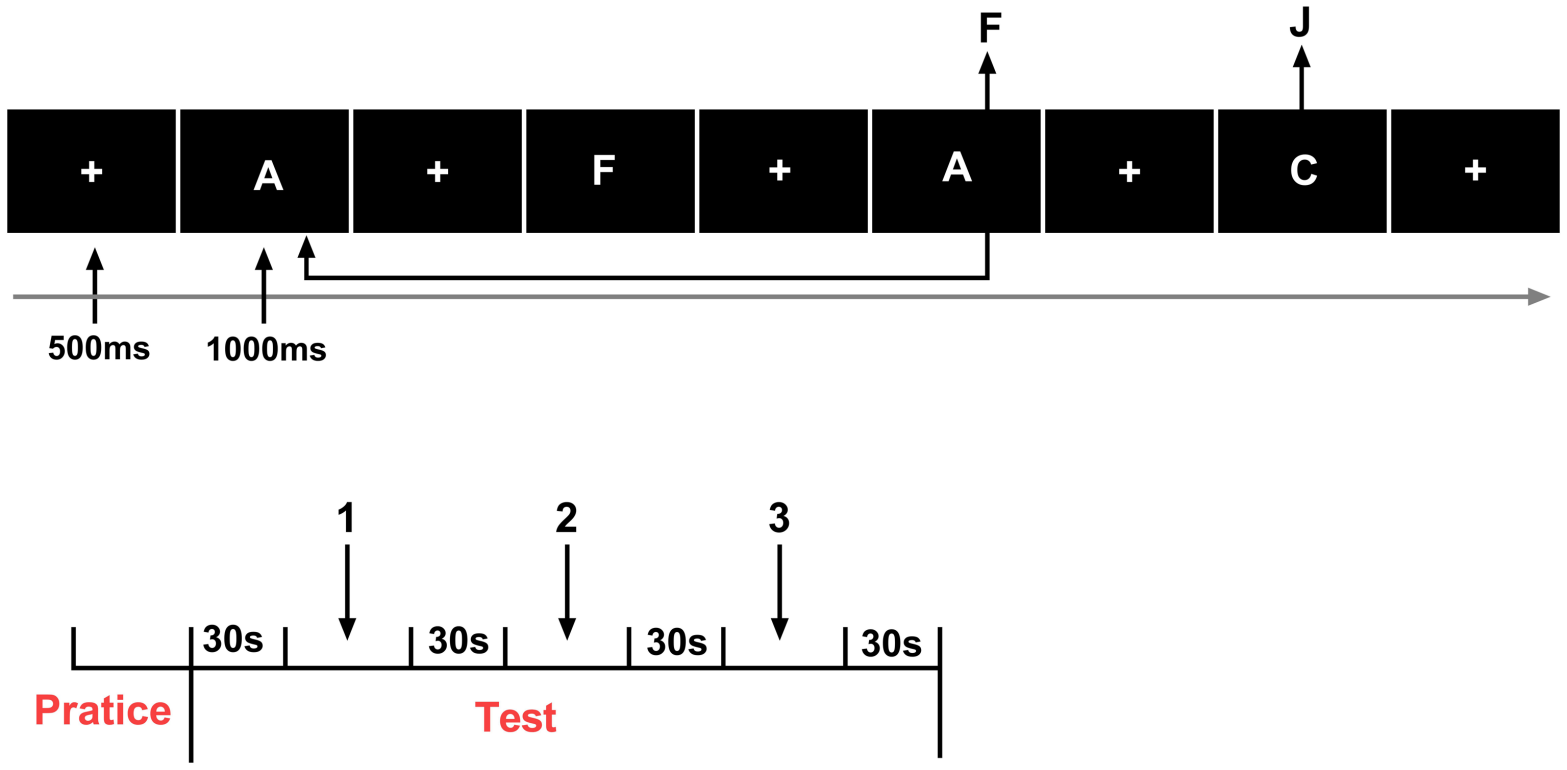
Schematic of the 2-Back task procedure.

The 2-Back task evaluated working memory performance. A continuous sequence of letters was presented one at a time, and participants were instructed to respond whenever the current stimulus matched the one presented two trials earlier. Behavioral indicators included RT and accuracy, reflecting the speed and precision of information updating and maintenance in working memory (Figure 5).

### 2.7 fNIRS data preprocessing

The fNIRS data were analyzed using the NirSpark software package. The preprocessing pipeline consisted of five main steps: (1) channels exhibiting excessive movement artifacts, signal dropout, or low signal quality across the entire recording were discarded; contaminated segments were corrected using spline interpolation or removed if necessary. Motion artifacts were identified through both visual inspection and automated detection in NirSpark; (2) conversion of raw light intensity data into optical density values; (3) application of a 0.01–0.2 Hz band-pass filter to suppress high-frequency noise and low-frequency drift (Li et al., 2013); (4) conversion of optical density values into relative concentration changes of HbO_2_ and deoxygenated hemoglobin (HbR) based on the modified Beer–Lambert law (Wang et al., 2020); (5) setting the time window for the hemodynamic response function (HRF), with a start time of −2 s and an end time of 102 s. In this setup, −2 to 0 s was defined as the baseline period, while 0–102 s was designated as the task block response period. Finally, the hemoglobin signals from each task block were superimposed and averaged to produce block-averaged responses for subsequent statistical analysis.

Noisy or invalid channels were identified and excluded based on the following criteria: (1) poor probe contact: Channels showing degraded signal quality or dropout due to unstable optode contact or improper probe placement; (2) excessive motion artifacts: Channels contaminated by substantial head or facial muscle movement, identified via both visual inspection and automated detection in NirSpark; (3) the automated detection used a moving-standard-deviation (MSD) algorithm that flagged abrupt signal fluctuations exceeding 5 standard deviations from the local mean; (4) high signal noise: channels where the light intensity deviated by more than 3 SD from the mean for over 20% of the total recording time. (5) Technical failures: channels affected by hardware malfunctions or sensor disconnection; (6) contaminated signal segments were corrected using spline interpolation or removed if necessary. These preprocessing steps ensured that only high-quality, artifact-free signals were retained for further analysis (Molavi and Dumont, 2010).

In this study, the mean change in ΔHbO_2_ during each task block was analyzed to assess prefrontal cortex activation across the Stroop, More–Odd Shifting, and 2-Back tasks. These paradigms are well established to engage the prefrontal cortex, where increased oxygen delivery is required to support cognitive processing. ΔHbO_2_ was chosen as the primary indicator of cortical activation because it provides a higher signal-to-noise ratio and greater sensitivity to task-related hemodynamic changes than ΔHbR (Hoshi, 2003). Although ΔHbO_2_ can be influenced by systemic physiological factors during exercise, such confounds were minimized through band-pass filtering (0.01–0.2 Hz) and baseline normalization relative to pre-task periods. Both increases and decreases in ΔHbO_2_ were interpreted in conjunction with behavioral outcomes. Specifically, an increase in ΔHbO_2_ accompanied by improved behavioral performance (positive feedback) was considered to reflect enhanced cortical activation and resource recruitment, whereas an increase associated with poorer performance (negative feedback) indicated reduced neural efficiency. Conversely, a decrease in ΔHbO_2_ coupled with improved performance reflected enhanced neural efficiency and optimized processing, while a decrease accompanied by behavioral decline suggested insufficient activation or suboptimal cortical engagement.

### 2.8 Statistical analysis

All data were analyzed using SPSS 27.0 software, with continuous variables reported as mean ± standard deviation. Before analysis, the variables’ normality and sphericity assumptions were tested. If sphericity was violated, the Greenhouse–Geisser correction was applied. In cases where the assumptions for parametric testing were severely violated, the Friedman test was used as a non-parametric alternative. The criteria for effect sizes were: Cohen’s *d* = 0.2, 0.5, 0.8 for small, medium, and large effects, respectively; and partial *η*^2^ = 0.010, 0.059, 0.138 for small, medium, and large effects, respectively. Statistical significance was set at *p* < 0.05.

#### 2.8.1 Sample size estimation

The sample size estimation was carried out using G*Power 3.1 software. Based on an effect size of *f* = 0.4, an alpha level (*α*) of 0.05, and a desired power (1 − *β*) of 0.95, this indicated a minimum *N* = 18; therefore, we enrolled *N* = 24 to increase precision and safeguard against attrition (no withdrawals occurred).

#### 2.8.2 Behavioral analysis

Before examining the effects of exercise intensity on cognitive performance, baseline equivalence across the three intensity conditions (30%, 50%, and 70% MVC) was evaluated. Pre-test data for accuracy and reaction time from each cognitive task (Stroop, More–Odd Shifting, and 2-back) were analyzed using a one-way repeated-measures ANOVA. This preliminary analysis confirmed that there were no significant baseline differences among conditions (all *p* > 0.05), indicating that participants started each intervention with comparable cognitive performance. For the behavioral data, a two-way repeated-measures ANOVA was conducted with time (pre-test vs. post-test) and intensity (30%, 50%, and 70% MVC) as within-subject factors, to examine performance changes in accuracy and RT. When significant main effects or interactions were found, Bonferroni-corrected *post-hoc* comparisons were performed to identify differences between intensity conditions and time points.

#### 2.8.3 fNIRS analysis

Statistical analyses of the fNIRS data focused on changes in concentration HbO_2_ within predefined ROIs across the PFC during the cognitive tasks. All analyses were performed at the ROI level, and channel-wise analyses were not conducted in the final version of the study to maintain analytical focus and readability. Statistical analyses of the fNIRS data focused on changes in HbO_2_ within the PFC during the task paradigm. A two-way repeated-measures ANOVA was performed for each ROIs, with time (pre-test vs. post-test) and intensity (30%, 50%, and 70% MVC) as within-subject factors. Three *F*-values were obtained for each ROIs, representing the main effects of time, intensity, and their interaction (time × intensity). Across ROIs, *p*-values for the time × intensity interaction (and, where applicable, simple effects) were corrected for multiple comparisons using the false discovery rate (FDR) procedure (Benjamini–Hochberg). Results were considered statistically significant at *p* < 0.05 after FDR correction. When a significant time × intensity interaction was detected, individual change scores (ΔHbO_2_ = post − pre) were calculated. These values were then compared using a one-way repeated-measures ANOVA with intensity (30%, 50%, and 70% MVC) as the within-subject factor, followed by Bonferroni-adjusted pairwise comparisons where significant omnibus effects were found. In parallel, simple effects of time were examined within each intensity level using paired-sample *t*-tests to determine whether HbO_2_ signals changed significantly from pre- to post-test. When the time × intensity interaction was non-significant or when the corrected *p*-value exceeded 0.05, intensity effects were not further interpreted.

## 3 Results

### 3.1 Behavioral findings

To confirm baseline equivalence, pre-test behavioral data from the three intensity conditions were analyzed using a one-way ANOVA. The analysis revealed no significant differences in either accuracy or RT between conditions, confirming that all participants began the interventions with comparable cognitive baselines. All data satisfied the normality assumption (Shapiro–Wilk test, *p* > 0.05). Where Mauchly’s test indicated a violation of sphericity, the Greenhouse–Geisser correction was applied, and the adjusted degrees of freedom were reported as *F*(2,22) (Table 2).

**Table 2:** One-way ANOVA results for pre-intervention (baseline) performance across intensity groups in three cognitive paradigms.

Condition	30% MVC	50% MVC	70% MVC	*F*	*p*	*η* ^2^
Stroop task						
Congruent accuracy (%)	95.97 ± 2.87	94.70 ± 4.53	95.97 ± 3.37	0.912	0.409	0.038
Congruent RT (ms)	625.96 ± 76.74	617.67 ± 90.66	607.67 ± 97.41	0.271	0.764	0.012
Incongruent accuracy (%)	96.81 ± 2.77	96.02 ± 3.35	97.42 ± 4.05	1.025	0.375	0.085
Incongruent RT (ms)	636.54 ± 70.63	632.96 ± 109.26	610.04 ± 105.60	0.577	0.566	0.024
More-Odd Shifting task						
Switching accuracy (%)	91.71 ± 3.17	90.23 ± 5.81	93.31 ± 4.78	2.734	0.076	0.106
Switching RT (ms)	749.54 ± 108.34	800.08 ± 154.42	730.63 ± 96.30	2.269	0.115	0.090
Non-switching accuracy (%)	93.86 ± 3.37	92.28 ± 5.79	93.91 ± 4.72	1.093	0.344	0.045
Non-switching RT (ms)	689.96 ± 125.52	662.04 ± 123.07	648.71 ± 86.62	1.039	0.362	0.043
2-Back task						
Accuracy (%)	88.81 ± 5.73	86.18 ± 7.19	89.24 ± 5.91	1.449	0.245	0.059
Reaction Time (ms)	673.13 ± 152.24	699.79 ± 163.73	664.75 ± 168.73	0.387	0.682	0.017

Data are presented as Mean ± SD. MVC, Maximal voluntary contraction; RT, Reaction Time.

#### 3.1.1 Stroop task results

For the congruent condition, accuracy improved significantly over time (*p* < 0.05), but the effects of Intensity and the Time × Intensity interaction were nonsignificant (*p* > 0.05). RT showed a significant main effect of Time [*F*(1,23) = 15.300, *p* < 0.001, *η*^2^ = 0.399] and a significant Time × Intensity interaction [*F*(2,22) = 7.609, *p* = 0.003, *η*^2^ = 0.409]. *Post-hoc* tests revealed that the 50% intensity group had a greater RT reduction compared to the 30% and 70% groups (*p* < 0.01).

For the incongruent condition, a significant Time × Intensity interaction was found [*F*(2,46) = 6.058, *p* = 0.005, *η*^2^ = 0.208], with accuracy showing a marginal main effect of Time [*F*(1,23) = 3.986, *p* = 0.058, *η*^2^ = 0.148]. *Post-hoc* analyses indicated that accuracy improved most under the 50% intensity condition (*p* < 0.01) (Table 3).

**Table 3:** Results of two-way repeated-measures ANOVA for Stroop task performance across different intensity conditions.

Group	Pre-test	Post-test	
Congruent accuracy (%)			
30%MVC	95.97 ± 2.87	96.95 ± 2.64	
50%MVC	94.70 ± 4.53	96.69 ± 3.55^**^	
70%MVC	95.97 ± 3.38	95.68 ± 4.34	
	*F*_Time_(1,23) = 5.438	*p* = 0.029	*η*^2^ = 0.191
	*F*_Intensity_(2,46) = 0.409	*p* = 0.667	*η*^2^ = 0.017
	*F*_Time × Intensity_(2,22) = 1.496	*p* = 0.246	*η*^2^ = 0.120
Congruent RT (ms)			
30%MVC	625.96 ± 76.74	603.29 ± 72.56	
50%MVC	617.67 ± 90.66	568.71 ± 90.04^***^	
70%MVC	607.67 ± 97.41	600.21 ± 94.73	
	*F*_Time_(1,23) = 15.300	*p* < 0.001	*η*^2^ = 0.399
	*F*_Intensity_(2,46) = 0.425	*p* = 0.656	*η*^2^ = 0.018
	*F*_Time × Intensity_(2,22) = 7.609	*p* = 0.003	*η*^2^ = 0.409
Incongruent accuracy (%)			
30%MVC	96.82 ± 2.77	97.52 ± 2.33	
50%MVC	96.02 ± 3.35	98.16 ± 2.44^***^	
70%MVC	97.42 ± 4.05	96.82 ± 3.86	
	*F*_Time_(1,23) = 3.986	*p* = 0.058	*η*^2^ = 0.148
	*F*_Intensity_(2,22) = 0.011	*p* = 0.989	*η*^2^ = 0.001
	*F*_Time × Intensity_(2,46) = 6.058	P = 0.005	*η*^2^ = 0.208
Incongruent RT (ms)			
30%MVC	636.54 ± 70.63	602.54 ± 71.81^*^	
50%MVC	632.96 ± 109.25	592.33 ± 103.30^***^	
70%MVC	610.04 ± 105.60	596.00 ± 83.08	
	*F*_Time_(1,23) = 21.580	*p* < 0.001	*η*^2^ = 0.484
	*F*_Intensity_(2,46) = 0.228	*p* = 0.797	*η*^2^ = 0.010
	*F*_Time × Intensity_(2,46) = 1.281	*p* = 0.287	*η*^2^ = 0.053

Data are presented as Mean ± SD. RT, Reaction Time.*p < 0.05 (post-test vs. pre-test); **p < 0.01; ***p < 0.001.

#### 3.1.2 More–Odd Shifting task results

For the More–Odd Shifting task, in switching trials, accuracy showed a significant main effect of Time [*F*(1,23) = 78.071, *p* < 0.001, *η*^2^ = 0.772], indicating improved performance after intervention. Intensity had no significant main effect (*p* > 0.05), but the Time × Intensity interaction was significant [*F*(2,22) = 3.319, *p* = 0.045, *η*^2^ = 0.126]. *Post-hoc* comparisons revealed that the 50% intensity group showed the most significant accuracy improvement. For RT, there was a strong main effect of Time [*F*(1,23) = 128.782, *p* < 0.001, *η*^2^ = 0.848] and a significant Time × Intensity interaction [*F*(2,46) = 3.739, *p* = 0.031, *η*^2^ = 0.140]. *Post-hoc* analyses indicated that RT reduction was most pronounced under the 50% intensity condition (*p* < 0.05).

In non-switching trials, accuracy showed a significant main effect of Time [*F*(1,23) = 35.569, *p* < 0.001, *η*^2^ = 0.614] and a significant Time × Intensity interaction [*F*(2,46) = 5.064, *p* = 0.010, *η*^2^ = 0.180], with no significant main effect of Intensity (*p* > 0.05). *Post-hoc* comparisons again indicated the 50% intensity group showed the most improvement in accuracy. RT for non-switching trials displayed a robust main effect of Time [*F*(1,23) = 49.742, *p* < 0.001, *η*^2^ = 0.684], with faster responses after training, but the effects of Intensity and interaction were nonsignificant (*p* > 0.05) (Table 4).

**Table 4:** Results of two-way repeated-measures ANOVA for More-Odd Shifting task performance across different intensity conditions.

Group	Pre-test	Post-test	
Switching accuracy (%)			
30%MVC	91.71 ± 3.17	94.44 ± 2.62^***^	
50%MVC	90.23 ± 5.81	94.73 ± 3.39^***^	
70%MVC	93.31 ± 4.78	95.43 ± 2.92^*^	
	*F*_Time_(1,23) = 78.071	*p* < 0.001	*η*^2^ = 0.772
	*F*_Intensity_(2,46) = 1.886	*p* = 0.163	*η*^2^ = 0.076
	*F*_Time × Intensity_(2,22) = 3.319	*p* = 0.045	*η*^2^ = 0.126
Switching RT (ms)			
30%MVC	749.54 ± 108.34	682.79 ± 94.33^***^	
50%MVC	800.08 ± 154.42	679.58 ± 109.70^***^	
70%MVC	730.62 ± 96.30	655.13 ± 80.32^***^	
	*F*_Time_(1,23) = 128.782	*p* < 0.001	*η*^2^ = 0.848
	*F*_Intensity_(2,46) = 1.415	*p* = 0.253	*η*^2^ = 0.058
	*F*_Time × Intensity_(2,46) = 3.739	*p* = 0.031	*η*^2^ = 0.140
Non-switching accuracy (%)			
30%MVC	93.86 ± 3.37	96.96 ± 3.71^**^	
50%MVC	92.70 ± 5.75	96.28 ± 3.71^***^	
70%MVC	93.91 ± 4.72	94.69 ± 3.76	
	*F*_Time_(1,23) = 35.569	*p* < 0.001	*η*^2^ = 0.614
	*F*_Intensity_(2,22) = 1.367	*p* = 0.276	*η*^2^ = 0.111
	*F*_Time × Intensity_(2,46) = 5.064	*p* = 0.010	*η*^2^ = 0.180
Non-switching RT (ms)			
30%MVC	689.96 ± 125.52	625.75 ± 119.99^*^	
50%MVC	662.04 ± 123.07	565.95 ± 98.12^***^	
70%MVC	648.71 ± 86.62	585.38 ± 100.94^***^	
	*F*_Time_(1,23) = 49.742	*p* < 0.001	*η*^2^ = 0.684
	*F*_Intensity_(2,46) = 1.562	*p* = 0.221	*η*^2^ = 0.064
	*F*_Time × Intensity_(2,46) = 1.342	*p* = 0.271	*η*^2^ = 0.055

Data are presented as Mean ± SD. RT, Reaction Time. *p < 0.05 (post-test vs. pre-test); **p < 0.01; ***p < 0.001.

#### 3.1.3 2-Back task results

For the 2-Back task, accuracy showed a significant main effect of Time [*F*(1,23) = 31.257, *p* < 0.001, *η*^2^ = 0.576], indicating overall improvement after the intervention. However, neither the main effect of Intensity (*p* > 0.05) nor the Time × Intensity interaction (*p* > 0.05) reached significance.

**Figure 6:**
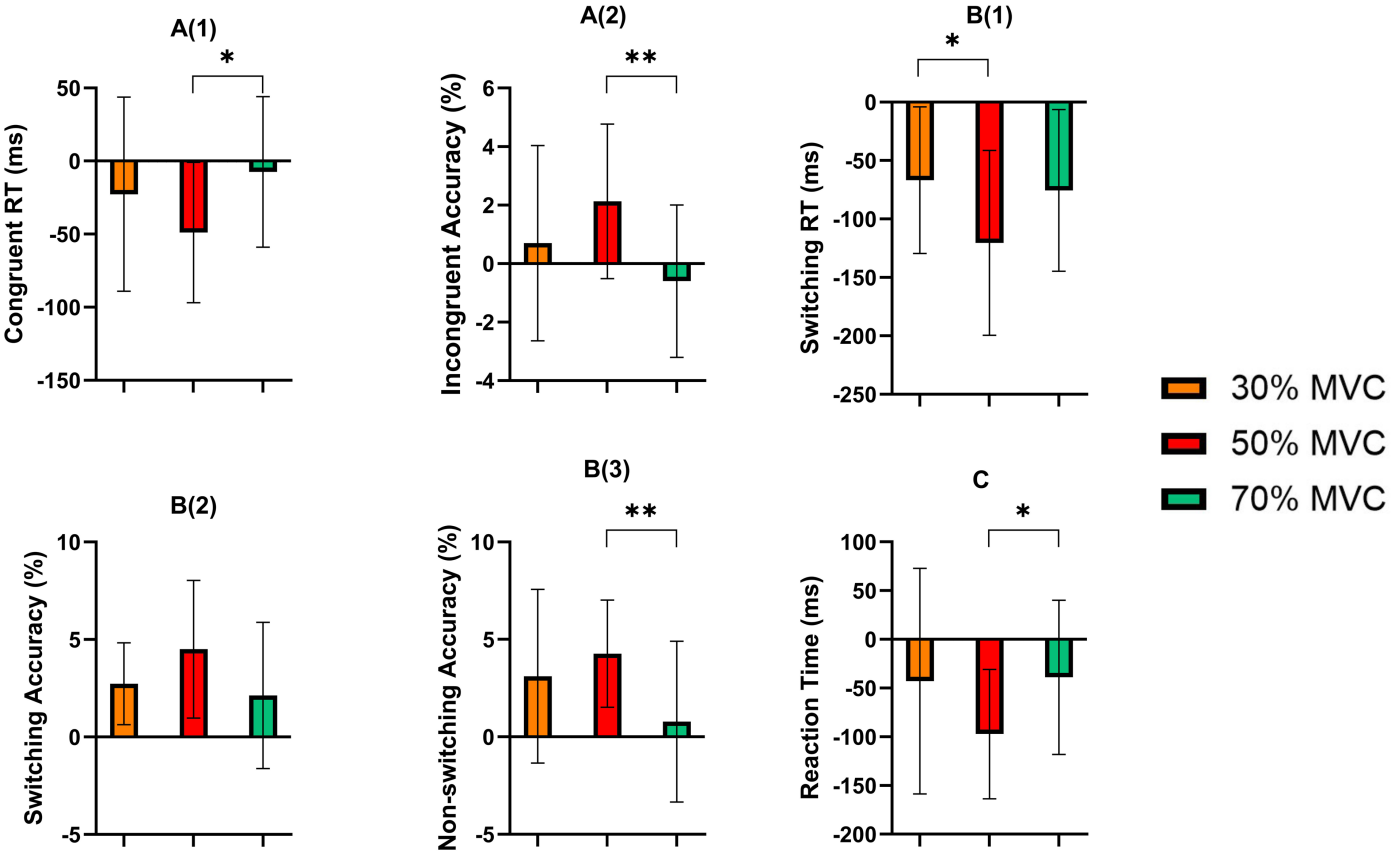
One-way ANOVA and *post-hoc* comparison results for tasks performance indicators. **A(1)** Stroop task performance Congruent Reaction Time (ms); **A(2)** Stroop task performance Incongruent Accuracy (%); **B(1)** More-Odd Shifting task performance Switching Reaction Time (ms); **B(2)** More-Odd Shifting task performance Switching Accuracy (%); **B(3)** More-Odd Shifting task performance Non-switching Accuracy (%); **(C)** 2-Back task performance Reaction Time (ms). *p < 0.05; **p < 0.01. Error bars indicate standard deviation of the mean (SD).

For RT, a strong main effect of Time was observed [*F*(1,23) = 26.373, *p* < 0.001, *η*^2^ = 0.534], reflecting significantly faster responses at post-test. Although the main effect of Intensity was nonsignificant (*p* > 0.05), a significant Time × Intensity interaction emerged [*F*(2,46) = 3.543, *p* = 0.037, *η*^2^ = 0.133]. *Post-hoc* comparisons revealed that the 50% intensity group showed a greater RT reduction compared to both the 30% and 70% intensity groups (*p* < 0.05) (Table 5; Figure 6).

**Table 5:** Results of two-way repeated-measures ANOVA for 2-Back task performance across different intensity conditions.

Group	Pre-test	Post-test	
Accuracy (%)			
30%MVC	88.81 ± 5.73	90.93 ± 3.79^*^	
50%MVC	86.18 ± 7.19	88.93 ± 5.93^**^	
70%MVC	89.24 ± 5.92	91.86 ± 4.13^**^	
	*F*_Time_(1,23) = 31.257	*p* < 0.001	*η*^2^ = 0.576
	*F*_Intensity_(2,46) = 1.950	*p* = 0.154	*η*^2^ = 0.078
	*F*_Time × Intensity_(2,46) = 0.153	*p* = 0.858	*η*^2^ = 0.007
Reaction Time (ms)			
30%MVC	640.67 ± 118.82	597.79 ± 124.42	
50%MVC	699.79 ± 163.73	602.58 ± 152.81^***^	
70%MVC	652.25 ± 159.82	613.29 ± 120.54^*^	
	*F*_Time_(1,23) = 26.373	*p* < 0.001	*η*^2^ = 0.534
	*F*_Intensity_(2,46) = 0.412	*p* = 0.665	*η*^2^ = 0.018
	*F*_Time × Intensity_(2,46) = 3.543	*p* = 0.037	*η*^2^ = 0.133

Data are presented as Mean ± SD. RT, Reaction Time. *p < 0.05 (post-test vs. pre-test); **p < 0.01; ***p < 0.001.

### 3.2 fNIRS results

All data satisfied the normality assumption (Shapiro–Wilk test, *p* > 0.05). Where Mauchly’s test indicated a violation of sphericity, the Greenhouse–Geisser correction was applied, and the adjusted degrees of freedom were reported as *F*(2,22).

#### 3.2.1 Hemodynamic activity across RIOs in the Stroop task

**Figure 7:**
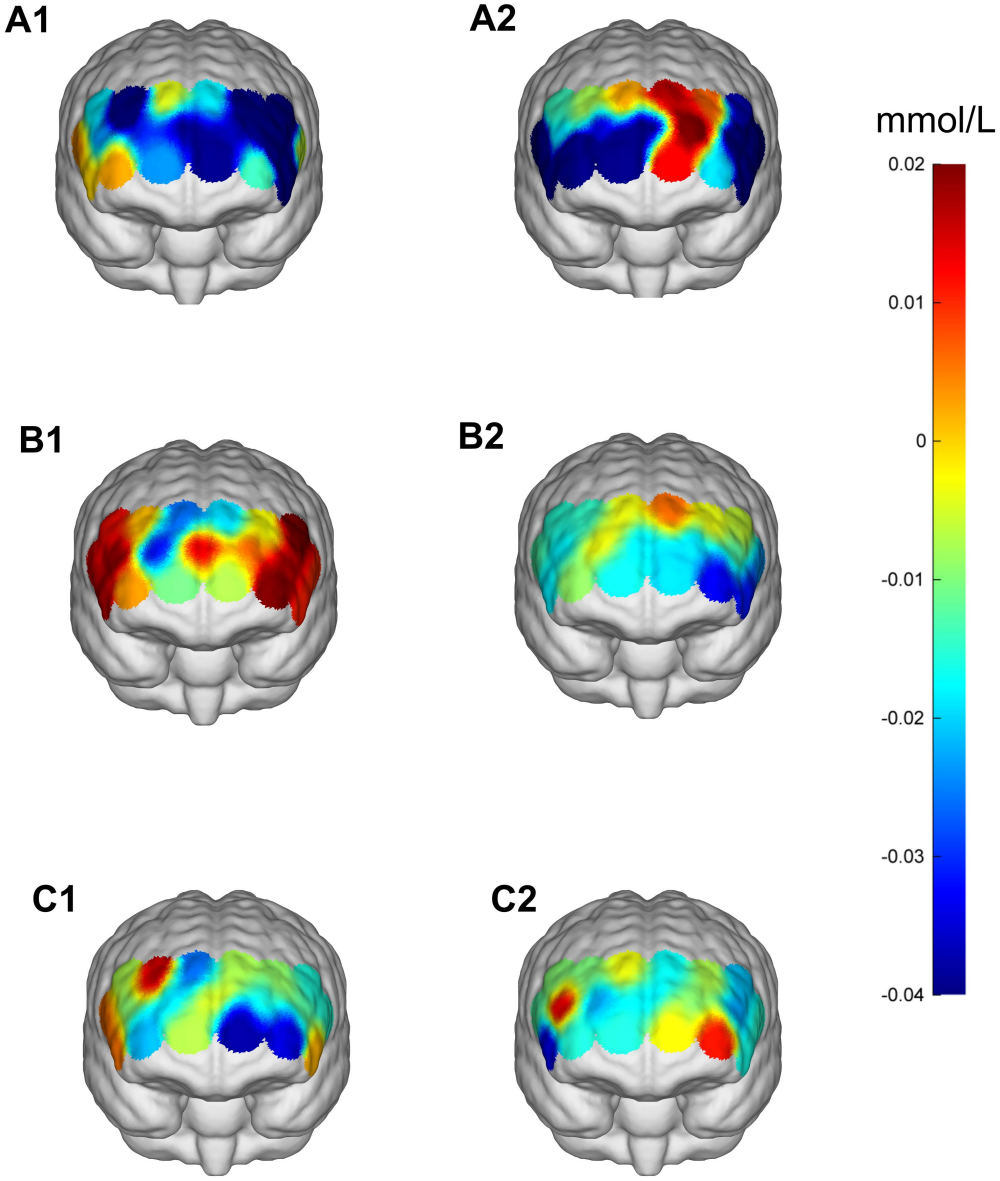
Prefrontal cortex ∆HbO_2_ (mmol/L) changes during the Stroop task. **(A1,A2)** 30% maximal voluntary contraction (MVC) pre/post; **(B1,B2)** 50% maximal voluntary contraction (MVC) pre/post; **(C1,C2)** 70% maximal voluntary contraction (MVC) pre/post. ΔHbO_2,_ change in oxygenated hemoglobin concentration.

**Figure 8:**
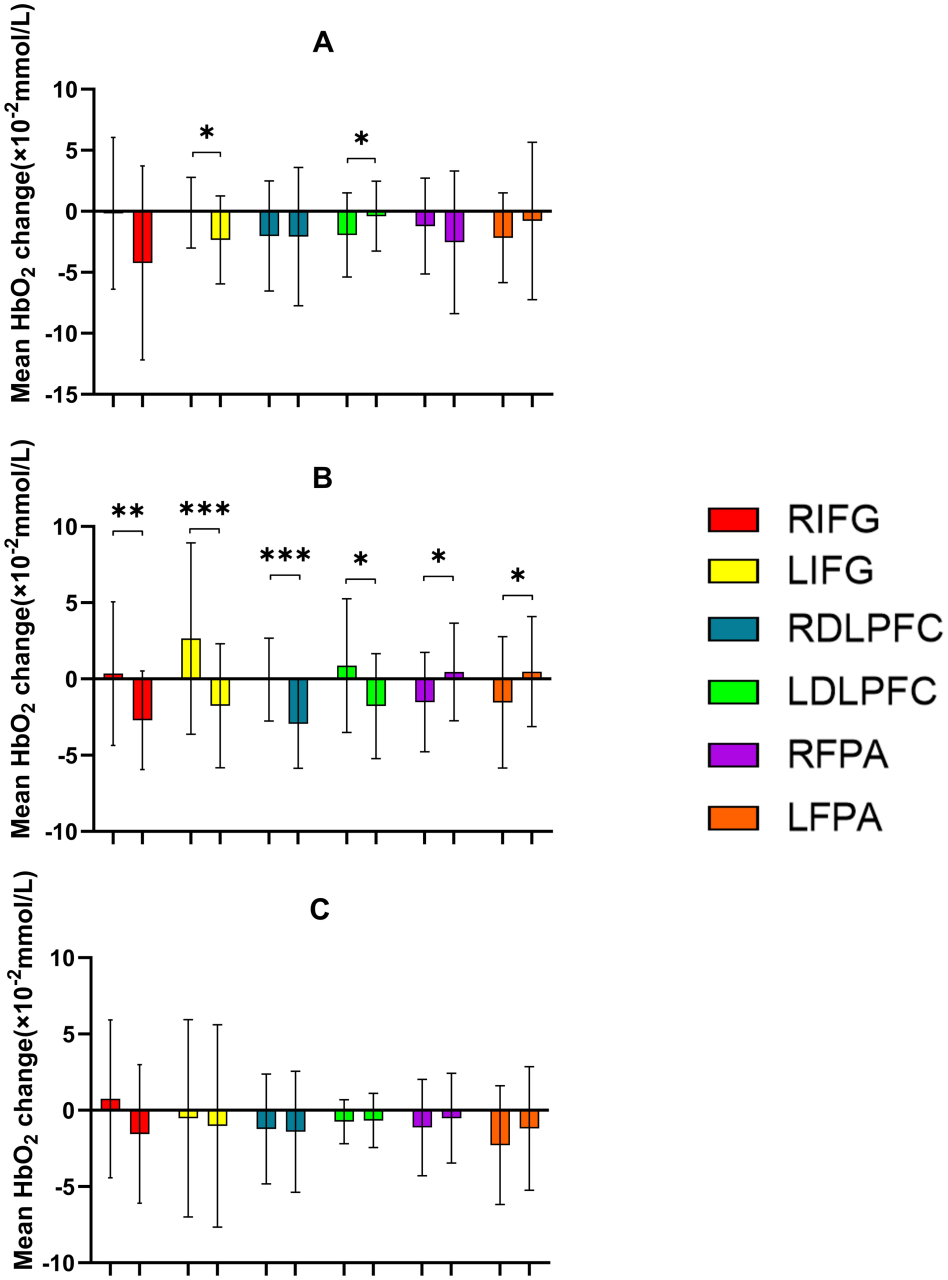
Comparison of HbO_2_ changes across different RIOs in the prefrontal cortex before and after the Stroop task. **(A)** 30% maximal voluntary contraction (MVC) pre/post; **(B)** 50% MVC pre/post; **(C)** 70% MVC pre/post. *Significant difference between post-and pre-intervention, *p* < 0.05; **, *p* < 0.01. ***, *p* < 0.001. Error bars indicate standard deviation of the mean (SD).

During the Stroop task paradigm, a significant main effect of Time was found in RIFG [*F*(1,23) = 9.303, *p* = 0.006, *η*^2^ = 0.288] and LIFG [*F*(1,23) = 13.220, *p* = 0.001, *η*^2^ = 0.365], indicating enhanced cortical activation after intervention. Significant Time × Intensity interactions were observed in multiple ROIs, including LIFG [*F*(2,46) = 3.577, *p* = 0.036, *η*^2^ = 0.135], RDLPFC [*F*(2,22) = 3.575, *p* = 0.036, *η*^2^ = 0.135], LDLPFC [*F*(2,22) = 5.975, *p* = 0.008, *η*^2^ = 0.352], RFPA [*F*(2,46) = 4.282, *p* = 0.020, *η*^2^ = 0.157], and LFPA [*F*(2,46) = 4.130, *p* = 0.022, *η*^2^ = 0.152]. *Post-hoc* analyses revealed that the 50% intensity condition produced the most distinct decrease in HbO_2_, showing significantly greater decreases in LIFG, RDLPFC, and LDLPFC (all *p* = 0.043, FDR-corrected) compared with both the 30 and 70% conditions, indicating more efficient hemodynamic regulation under moderate stimulation. Similar patterns were also observed in RFPA and LFPA (Tables 6, 7; Figures 7, 8).

**Table 6:** Two-way repeated-measures ANOVA results for changes in HbO_2_ (×10^−2^ mmol/L) across ROIs during the Stroop task under different intensity conditions.

Group	Pre-test	Post-test	
Right inferior prefrontal gyrus			
30%MVC	−0.17 ± 6.22	−4.23 ± 7.95	
50%MVC	0.35 ± 4.71	−2.71 ± 3.24^**^	
70%MVC	0.75 ± 5.18	−1.55 ± 4.54	
	*F*_Time_(1,23) = 9.303	*p* = 0.006	*η*^2^ = 0.288
	*F*_Intensity_(2,46) = 1.297	*p* = 0.283	*η*^2^ = 0.053
	*F*_Time × Intensity_(2,22) = 0.389	*p* = 0.682	*η*^2^ = 0.034
Left inferior prefrontal gyrus			
30%MVC	−0.12 ± 2.90	−2.34 ± 3.60^**^	
50%MVC	2.65 ± 6.27	−1.76 ± 4.06^***^	
70%MVC	−0.53 ± 6.47	−1.02 ± 6.63	
	*F*_Time_(1,23) = 13.220	*p* = 0.001	*η*^2^ = 0.365
	*F*_Intensity_(2,46) = 0.810	*p* = 0.451	*η*^2^ = 0.034
	*F*_Time × Intensity_(2,46) = 3.577	*p* = 0.036	*η*^2^ = 0.135
Right dorsolateral prefrontal cortex			
30%MVC	−2.03 ± 4.51	−2.08 ± 5.67	
50%MVC	−0.04 ± 2.72	−2.95 ± 2.91^***^	
70%MVC	−1.22 ± 3.60	−1.41 ± 3.97	
	*F*_Time_(1,23) = 2.804	*p* = 0.108	*η*^2^ = 0.109
	*F*_Intensity_(2,46) = 4.003	*p* = 0.057	*η*^2^ = 0.012
	*F*_Time × Intensity_(2,22) = 3.575	*p* = 0.036	*η*^2^ = 0.135
Left dorsolateral prefrontal cortex			
30%MVC	−1.94 ± 3.44	−0.40 ± 2.87^*^	
50%MVC	0.87 ± 4.38	−1.78 ± 3.44^*^	
70%MVC	−0.75 ± 1.44	−0.67 ± 1.78	
	*F*_Time_(1,23) = 0.424	*p* = 0.521	*η*^2^ = 0.018
	*F*_Intensity_(2,22) = 0.766	*p* = 0.477	*η*^2^ = 0.065
	*F*_Time × Intensity_(2,22) = 5.975	*p* = 0.008	*η*^2^ = 0.352
Right frontopolar area			
30%MVC	−1.21 ± 3.93	−2.54 ± 5.84	
50%MVC	−1.52 ± 3.26	0.46 ± 3.20^*^	
70%MVC	−1.13 ± 3.16	−0.52 ± 2.94	
	*F*_Time_(1,23) = 0.498	*p* = 0.487	*η*^2^ = 0.021
	*F*_Intensity_(2,46) = 1.263	*p* = 0.292	*η*^2^ = 0.052
	*F*_Time × Intensity_(2,46) = 4.282	*p* = 0.020	*η*^2^ = 0.157
Left frontopolar area			
	−2.37 ± 3.67	−0.79 ± 6.45	
	−1.54 ± 4.31	0.48 ± 3.61^*^	
	−2.29 ± 3.89	−1.20 ± 4.05	
	*F*_Time_(1,23) = 0.051	*p* = 0.823	*η*^2^ = 0.002
	*F*_Intensity_(2,46) = 0.803	*p* = 0.454	*η*^2^ = 0.034
	*F*_Time × Intensity_(2,46) = 4.130	*p* = 0.022	*η*^2^ = 0.152

Data are presented as Mean ± SD. *p < 0.05 (post-test vs. pre-test); **p < 0.01; ***p < 0.001.

**Table 7:** One-way repeated-measures ANOVA results and post-hoc comparisons of ΔHbO_2_ across intensity levels during the Stroop task.

Group	ΔHbO_2_ (×10^−2^ mmol/L)
Left inferior prefrontal gyrus	
30%MVC	−2.22 ± 3.72
50%MVC	−4.41 ± 5.32
70%MVC	−0.50 ± 6.34
*p* = 0.043^*^ (Δ50% < Δ30%; Δ50% < Δ70%^*^; Δ30% < Δ70%)	
Right dorsolateral prefrontal cortex	
30%MVC	−0.06 ± 5.62
50%MVC	−2.91 ± 3.58
70%MVC	−0.19 ± 3.23
*p* = 0.043^*^ (Δ50% < Δ30%; Δ50% < Δ70%^*^: Δ70% < Δ30%)	
Left dorsolateral prefrontal cortex	
30%MVC	1.79 ± 3.45
50%MVC	−2.65 ± 5.14
70%MVC	0.08 ± 1.27
*p* = 0.043^*^ (Δ50% < Δ30%^**^; Δ50% < Δ70%^*^: Δ70% < Δ30%^*^)	
Right frontopolar area	
30%MVC	−1.39 ± 5.72
50%MVC	1.98 ± 3.64
70%MVC	0.61 ± 3.01
*p* = 0.043^*^ (Δ30% < Δ50%^*^; Δ70% < Δ50%: Δ30% < Δ70%)	
Left frontopolar area	
30%MVC	1.56 ± 5.65
50%MVC	−2.23 ± 4.96
70%MVC	1.09 ± 4.93
*p* = 0.043^*^ (Δ50% < Δ30%^*^; Δ50% < Δ70%: Δ70% < Δ30%)	

Data are presented as Mean ± SD. ΔHbO_2_ represents the change in oxyhemoglobin concentration (post–pre). The *p*-values were corrected for multiple comparisons using the false discovery rate (FDR) method. *p < 0.05; **p < 0.01.

#### 3.2.2 Hemodynamic activity across RIOs in the More–Odd Shifting task

**Figure 9:**
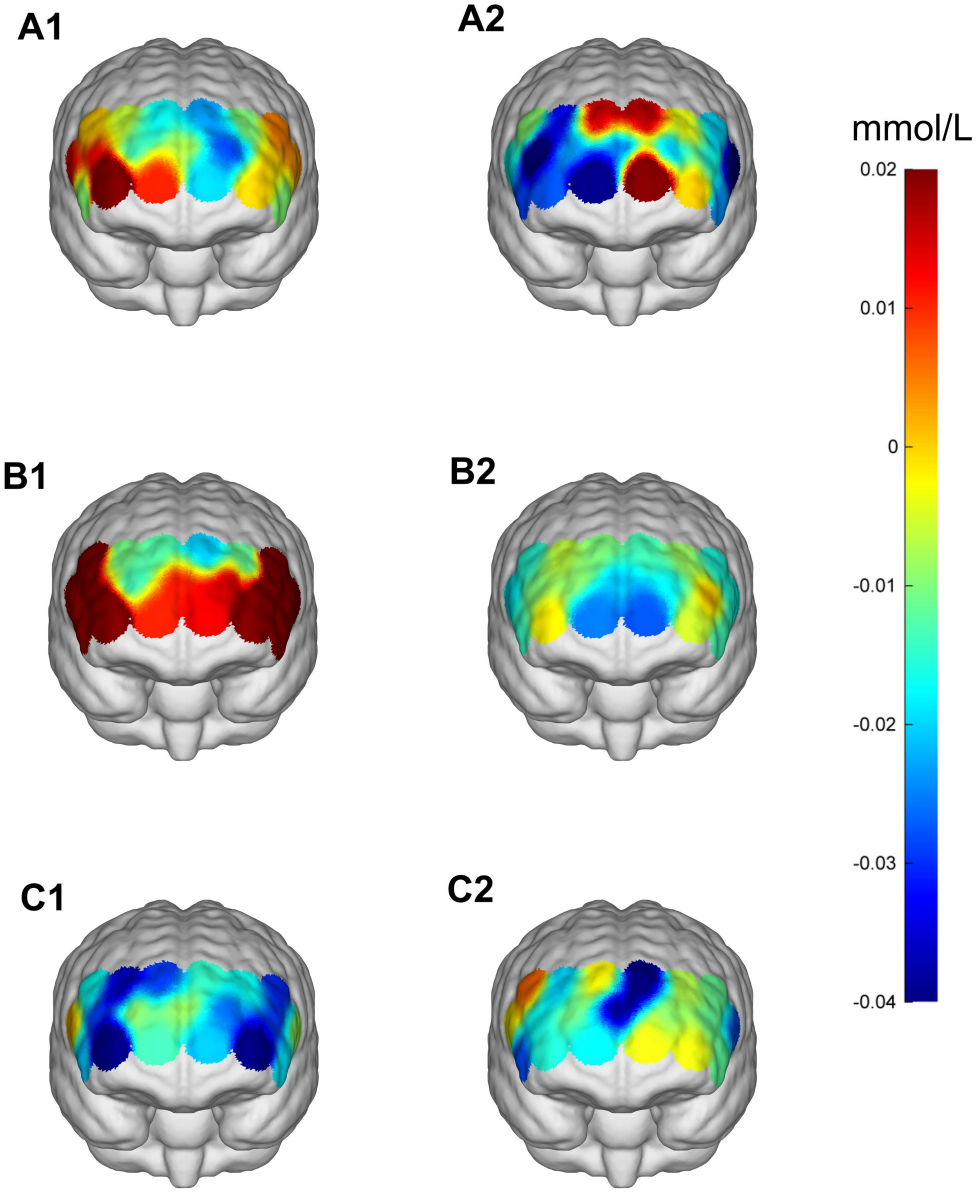
Prefrontal cortex ∆HbO_2_ (mmol/L) changes during the More-Odd Shifting task. **(A1,A2)** 30% maximal voluntary contraction (MVC) pre/post; **(B1,B2)** 50% maximal voluntary contraction (MVC) pre/post; **(C1,C2)** 70% maximal voluntary contraction (MVC) pre/post. ΔHbO_2,_ change in oxygenated hemoglobin concentration.

**Figure 10:**
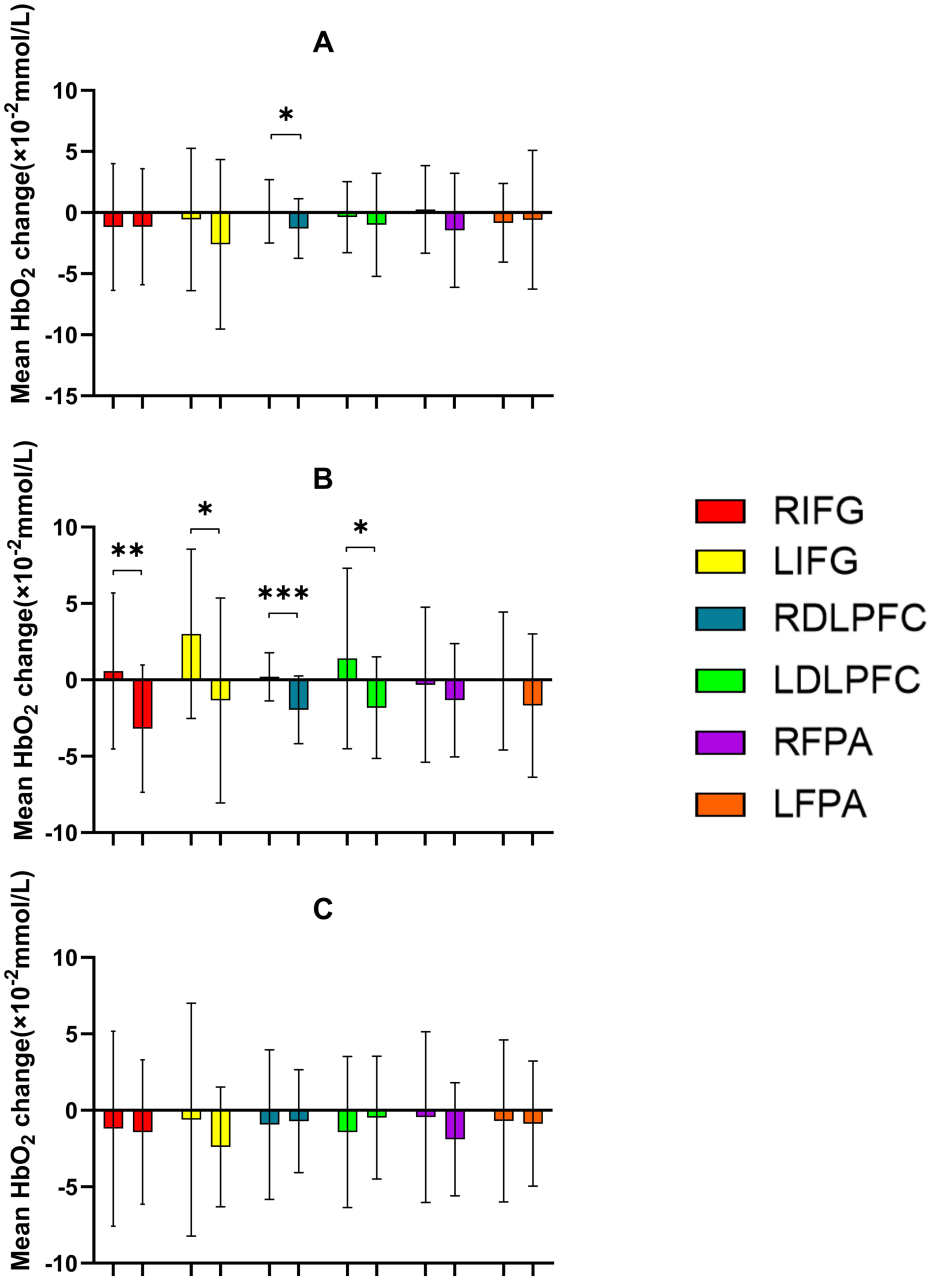
Comparison of HbO_2_ changes across different RIOs in the prefrontal cortex before and after the More-Odd Shifting task. **(A)** 30% maximal voluntary contraction (MVC) pre/post; **(B)** 50% MVC pre/post; **(C)** 70% MVC pre/post. *Significant difference between post-and pre-intervention, *p* < 0.05; **p < 0.01. ***p < 0.001. Error bars indicate standard deviation of the mean (SD).

During the Odd Shifting task. A significant main effect of Time was found in (LIFG) [*F*(1,23) = 9.223, *p* = 0.006, *η*^2^ = 0.286] and RDLPFC [*F*(1,23) = 13.587, *p* = 0.001, *η*^2^ = 0.371], indicating enhanced cortical activation over time following intervention. Additionally, significant Time × Intensity interactions were observed in RIFG [*F*(2,46) = 3.802, *p* = 0.030, *η*^2^ = 0.142], RDLPFC [*F*(2,46) = 3.242, *p* = 0.048, *η*^2^ = 0.124], LDLPFC [*F*(2,46) = 3.471, *p* = 0.039, *η*^2^ = 0.131], suggesting that the intensity of the intervention in these regions modulated the effect of time on cortical activation. However, no significant interaction was found in LFPA and RFPA (*p* > 0.05). Although no *post-hoc* comparisons reached significance after FDR correction (*p* > 0.05), the 50% intensity condition showed a trend toward improved neural efficiency (Table 8; Figures 9, 10).

**Table 8:** Two-way repeated-measures ANOVA results for changes in HbO_2_ (×10^−2^ mmol/L) across ROIs during the More–Odd Shifting task under different intensity conditions.

ROI	Pre-test	Post-test	
Right inferior prefrontal gyrus			
30%MVC	−1.17 ± 5.19	−1.16 ± 4.75	
50%MVC	0.58 ± 5.11	−3.19 ± 4.17^**^	
70%MVC	−1.20 ± 6.38	−1.42 ± 4.72	
	*F*_Time_(1,23) = 4.516	*p* = 0.045	*η*^2^ = 0.164
	*F*_Intensity_(2,46) = 0.010	*p* = 0.990	*η*^2^ = 0.000
	*F*_Time × Intensity_(2,46) = 3.802	*p* = 0.030^*^	*η*^2^ = 0.142
Left inferior prefrontal gyrus			
30%MVC	−0.56 ± 5.83	−2.60 ± 6.94	
50%MVC	3.01 ± 5.54	−1.35 ± 6.71^*^	
70%MVC	−0.61 ± 7.62	−2.39 ± 3.91	
	*F*_Time_(1,23) = 9.223	*p* = 0.006	*η*^2^ = 0.286
	*F*_Intensity_(2,46) = 2.058	*p* = 0.139	*η*^2^ = 0.082
	*F*_Time × Intensity_(2,46) = 0.984	*p* = 0.382	*η*^2^ = 0.410
Right dorsolateral prefrontal cortex			
30%MVC	0.10 ± 2.60	−1.30 ± 2.44^*^	
50%MVC	0.20 ± 1.57	−1.96 ± 2.21^***^	
70%MVC	−0.93 ± 4.89	−0.71 ± 3.36	
	*F*_Time_(1,23) = 13.587	*p* = 0.001^*^	*η*^2^ = 0.371
	*F*_Intensity_(2,22) = 0.064	*p* = 0.938	*η*^2^ = 0.003
	*F*_Time × Intensity_(2,46) = 3.242	*p* = 0.048^*^	*η*^2^ = 0.124
Left dorsolateral prefrontal cortex			
30%MVC	−0.37 ± 2.91	−1.00 ± 4.22	
50%MVC	1.40 ± 5.91	−1.82 ± 3.32^*^	
70%MVC	−1.42 ± 4.94	−0.48 ± 4.02	
	*F*_Time_(1,23) = 7.639	*p* = 0.011	*η*^2^ = 0.249
	*F*_Intensity_(2,46) = 0.189	*p* = 0.828	*η*^2^ = 0.008
	*F*_Time × Intensity_(2,46) = 3.471	*p* = 0.039^*^	*η*^2^ = 0.131
Right frontopolar area			
30%MVC	0.26 ± 3.58	−1.45 ± 4.66	
50%MVC	−0.32 ± 5.07	−1.33 ± 3.71	
70%MVC	−0.44 ± 5.59	−1.90 ± 3.70	
	*F*_Time_(1,23) = 5.300	*p* = 0.031	*η*^2^ = 0.187
	*F*_Intensity_(2,46) = 0.225	*p* = 0.799	*η*^2^ = 0.010
	*F*_Time × Intensity_(2,46) = 0.031	*p* = 0.970	*η*^2^ = 0.001
Left frontopolar area			
	−0.84 ± 3.22	−0.59 ± 5.68	
	−0.08 ± 4.52	−1.68 ± 4.69	
	−0.69 ± 5.30	−0.87 ± 4.09	
	*F*_Time_(1,23) = 0.774	*p* = 0.388	*η*^2^ = 0.033
	*F*_Intensity_(2,46) = 0.010	*p* = 0.990	*η*^2^ = 0.000
	*F*_Time × Intensity_(2,46) = 0.596	*p* = 0.555	*η*^2^ = 0.025

Data are presented as Mean ± SD. *p < 0.05(post-test vs. pre-test); **p < 0.01; ***p < 0.001.

#### 3.2.3 Hemodynamic activity across RIOs in the 2-Back task

**Figure 11:**
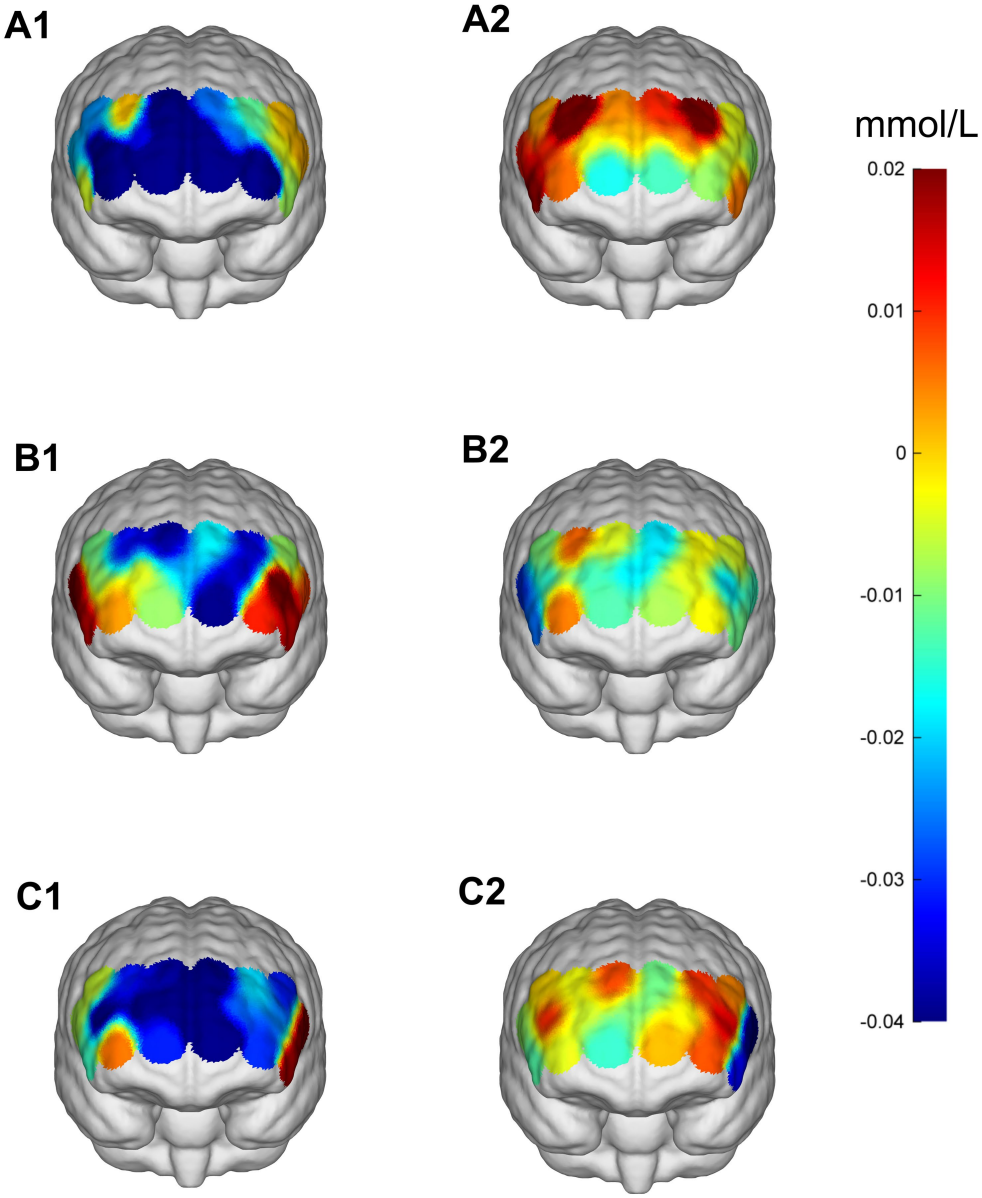
Prefrontal cortex ∆HbO_2_ (mmol/L) changes during the 2-Back task. **(A1,A2)** 30% maximal voluntary contraction (MVC) pre/post; **(B1,B2)** 50% maximal voluntary contraction (MVC) pre/post; **(C1,C2)** 70% maximal voluntary contraction (MVC) pre/post. ΔHbO_2,_ change in oxygenated hemoglobin concentration.

**Figure 12:**
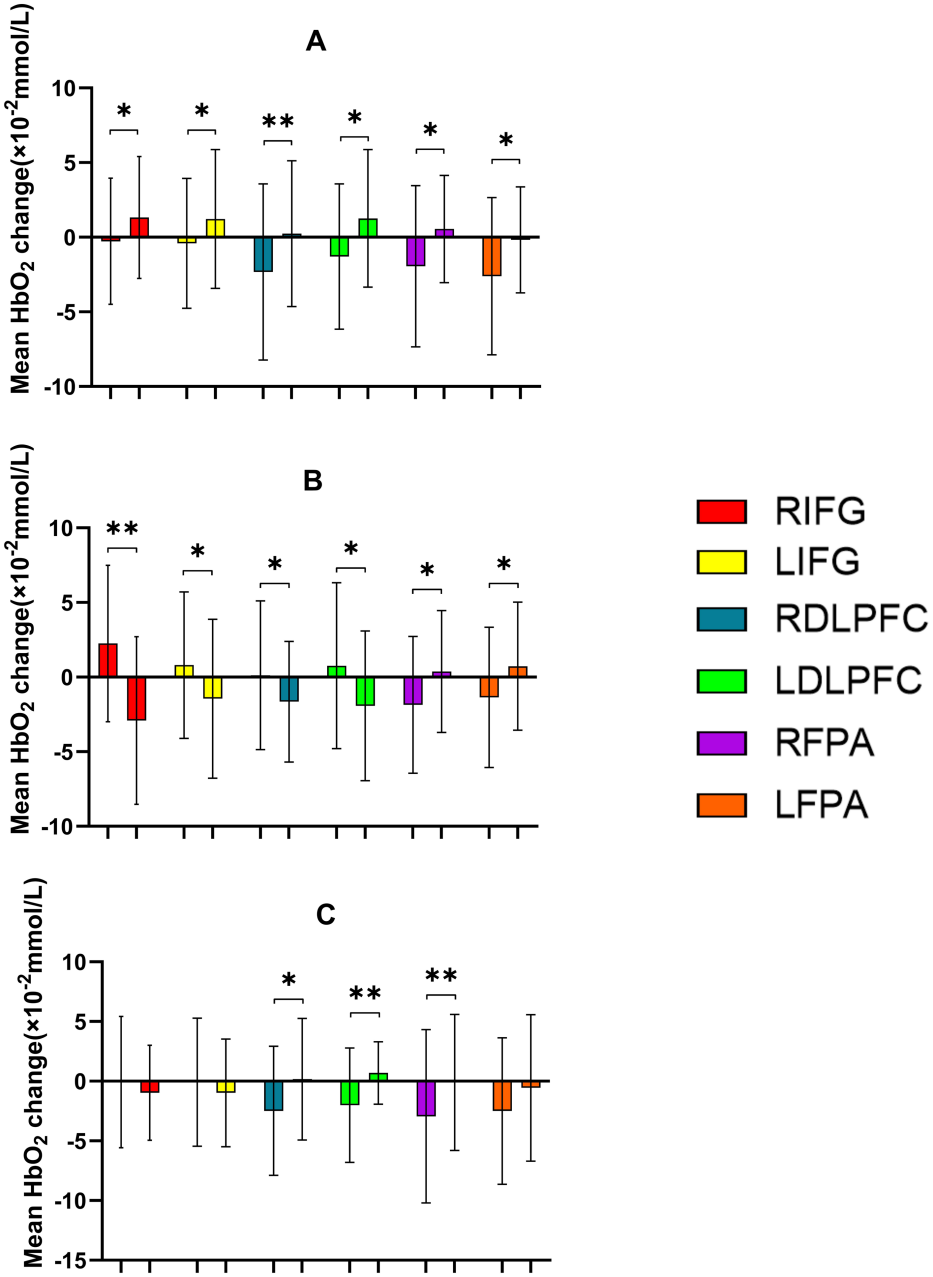
Comparison of HbO_2_ changes across different RIOs in the prefrontal cortex before and after the 2-Back task. **(A)** 30% maximal voluntary contraction (MVC) pre/post; **(B)** 50% MVC pre/post; **(C)** 70% MVC pre/post. *Significant difference between post-and pre-intervention, *p* < 0.05; **p < 0.01. Error bars indicate standard deviation of the mean (SD).

During the 2-Back task. A significant main effect of Time was observed in RIFG, RDLPFC, LFPA, and RFPA (*p* < 0.05), indicating increased cortical activation over time following intervention. However, no significant main effect of Time was found in LIFG or LDLPFC (*p* > 0.05). Significant Time × Intensity interactions were observed in RIFG [*F*(2,22) = 16.551, *p* < 0.001, *η*^2^ = 0.601], LIFG [*F*(2,46) = 4.460, *p* = 0.017, *η*^2^ = 0.162], RDLPFC [*F*(2,22) = 6.557, *p* = 0.003, *η*^2^ = 0.222], and LDLPFC [*F*(2,46) = 8.658, *p* < 0.001, *η*^2^ = 0.273], suggesting that the intensity of the intervention modulated the effect of time on cortical activation. No significant interactions were found in RFPA or LFPA (*p* > 0.05). *Post-hoc* analyses with FDR correction revealed that the 50% intensity condition resulted in significantly greater decreases in HbO_2_ compared to the 30% intensity condition in RIFG (*p* = 0.003), LIFG (*p* = 0.026), RDLPFC (*p* = 0.006), and LDLPFC (*p* = 0.003). The 50% intensity condition also showed greater reductions in ΔHbO_2_ than the 70% intensity in RIFG (*p* < 0.05) and RDLPFC (*p* < 0.01). In comparison, RDLPFC and LDLPFC exhibited significantly lower HbO_2_ with 30% intensity compared to 70% intensity (*p* < 0.05 for both regions). In conclusion, these findings suggest that moderate stimulation (50% intensity) optimally enhances neural efficiency and cortical activation in prefrontal regions, particularly in RIFG, LIFG, RDLPFC, and LDLPFC, during the 2-Back task (Tables 9, 10; Figures 11, 12).

**Table 9:** Two-way repeated-measures ANOVA results for changes in HbO_2_ (×10^−2^ mmol/L) across ROIs during the 2-Back task under different intensity conditions.

ROI	Pre-test	Post-test	
Right inferior prefrontal gyrus			
30%MVC	−0.27 ± 4.22	1.32 ± 4.08^*^	
50%MVC	2.25 ± 5.24	−2.91 ± 5.62^***^	
70%MVC	−0.07 ± 5.50	−0.97 ± 3.98	
	*F*_Time_(1,23) = 5.167	*p* = 0.033	*η*^2^ = 0.183
	*F*_Intensity_(2,22) = 1.017	*p* = 0.378	*η*^2^ = 0.085
	*F*_Time × Intensity_(2,22) = 16.551	*p* < 0.001	*η*^2^ = 0.601
Left inferior prefrontal gyrus			
30%MVC	−0.41 ± 4.35	1.23 ± 4.65^*^	
50%MVC	0.80 ± 4.91	−1.45 ± 5.32^*^	
70%MVC	−0.08 ± 5.37	−0.97 ± 4.51	
	*F*_Time_(1,23) = 0.548	*p* = 0.467	*η*^2^ = 0.023
	*F*_Intensity_(2,46) = 0.325	*p* = 0.724	*η*^2^ = 0.014
	*F*_Time × Intensity_(2,46) = 4.460	*p* = 0.017	*η*^2^ = 0.162
Right dorsolateral prefrontal cortex			
30%MVC	−2.32 ± 5.90	0.24 ± 4.88^**^	
50%MVC	0.12 ± 4.98	−1.65 ± 4.04^*^	
70%MVC	−2.48 ± 5.41	0.17 ± 5.09^*^	
	*F*_Time_(1,23) = 5.096	*p* = 0.034	*η*^2^ = 0.181
	*F*_Intensity_(2,46) = 0.040	*p* = 0.960	*η*^2^ = 0.002
	*F*_Time × Intensity_(2,22) = 6.557	*p* = 0.003	*η*^2^ = 0.222
Left dorsolateral prefrontal cortex			
30%MVC	−1.29 ± 4.87	1.26 ± 4.61^**^	
50%MVC	0.76 ± 5.56	−1.93 ± 5.02^*^	
70%MVC	−2.01 ± 4.79	0.69 ± 2.61^*^	
	*F*_Time_(1,23) = 2.207	*p* = 0.151	*η*^2^ = 0.088
	*F*_Intensity_(2,46) = 0.190	*p* = 0.828	*η*^2^ = 0.008
	*F*_Time × Intensity_(2,46) = 8.658	*p* < 0.001	*η*^2^ = 0.273
Right frontopolar area			
30%MVC	−1.94 ± 5.40	0.54 ± 3.59^*^	
50%MVC	−1.86 ± 4.59	0.37 ± 4.08^*^	
70%MVC	−2.94 ± 7.26	−0.10 ± 5.70^**^	
	*F*_Time_(1,23) = 19.943	*p* < 0.001	*η*^2^ = 0.464
	*F*_Intensity_(2,22) = 0.131	*p* = 0.878	*η*^2^ = 0.012
	*F*_Time × Intensity_(2,46) = 0.100	*p* = 0.905	*η*^2^ = 0.004
Left frontopolar area			
30%MVC	−2.61 ± 5.26	−0.18 ± 3.55^*^	
50%MVC	−1.36 ± 4.70	0.73 ± 4.29^*^	
70%MVC	−2.50 ± 6.13	−0.56 ± 6.13	
	*F*_Time_(1,23) = 16.358	*p* < 0.001	*η*^2^ = 0.416
	*F*_Intensity_(2,46) = 0.724	*p* = 0.490	*η*^2^ = 0.031
	*F*_Time × Intensity_(2,46) = 0.049	*p* = 0.952	*η*^2^ = 0.002

Data are presented as Mean ± SD. *p < 0.05 (post-test vs. pre-test); **p < 0.01; ***p < 0.001.

**Table 10:** One-way repeated-measures ANOVA results and post-hoc comparisons of ΔHbO_2_ across intensity levels during the 2-Back task.

RIO	Group	ΔHbO_2_ (×10^−2^ mmol/L)
Right inferior prefrontal gyrus		
30%MVC		1.59 ± 2.81
50%MVC		−5.16 ± 6.73
70%MVC		−0.89 ± 5.71
*p* = 0.003^**^ (Δ50% < Δ30%^***^; Δ50% < Δ70%^*^; Δ70% < Δ30%^*^)		
Left inferior prefrontal gyrus		
30%MVC		1.65 ± 3.59
50%MVC		−2.28 ± 4.98
70%MVC		−0.89 ± 6.26
*p* = 0.026^*^ (Δ50% < Δ30%^***^; Δ50% < Δ70%; Δ70% < Δ30%)		
Right dorsolateral prefrontal cortex		
30%MVC		2.56 ± 3.61
50%MVC		−1.78 ± 4.16
70%MVC		2.66 ± 5.94
*p* = 0.006^**^ (Δ50% < Δ30%^**^; Δ50% < Δ70%^*^; Δ30% < Δ70%)		
Left dorsolateral prefrontal cortex		
30%MVC		2.55 ± 4.52
50%MVC		−2.69 ± 6.21
70%MVC		2.70 ± 4.13
*p* = 0.003^**^ (Δ50% < Δ30%^*^; Δ50% < Δ70%^**^; Δ30% < Δ70%)		

Data are presented as Mean ± SD. ΔHbO_2_ represents the change in oxyhemoglobin concentration (post–pre). The *p*-values were corrected for multiple comparisons using the false discovery rate (FDR) method. *p < 0.05; **p < 0.01; ***p < 0.001.

## 4 Discussion

The primary aim of this study was to investigate the effects of rhythmic handgrip (RHG) exercise at varying intensities (30, 50, and 70% MVC) on cognitive function, specifically focusing on executive domains such as inhibitory control, cognitive flexibility, and working memory. The results demonstrate that moderate-intensity RHG (50% MVC) led to the most pronounced cognitive improvements, characterized by faster reaction times and higher accuracy across all three cognitive tasks. These behavioral gains were accompanied by enhanced neural efficiency in the PFC, as measured by fNIRS. Together, these findings suggest that moderate-intensity resistance-based exercise optimizes both behavioral and neural markers of executive functioning.

The observed cognitive benefits of moderate-intensity RHG align with the inverted-U model of cortical arousal, which posits that both insufficient and excessive levels of neural activation can impair cognitive performance (Sieck et al., 2016). Specifically, the 50% MVC condition produced the most efficient balance between activation and control, as indicated by faster responses and higher accuracy in Stroop, More–Odd Shifting, and 2-Back tasks. In contrast, low-intensity exercise (30% MVC) likely failed to elicit sufficient cortical engagement to improve cognitive control. In comparison, high-intensity RHG (70% MVC) induced an overload of neural activity, consistent with prior reports that excessive arousal impairs prefrontal-dependent tasks (Bherer et al., 2013; Brown et al., 2012). These results reinforce the idea that optimal cognitive outcomes arise from a moderate level of physiological and neural activation. The fNIRS findings provide further support for this conclusion. Moderate-intensity RHG induced reduced HbO_2_ concentrations in key prefrontal areas, including the dorsolateral prefrontal cortex DLPFC and IFG, regions critical for inhibitory control and cognitive flexibility. The decrease in HbO_2_ suggests that better task performance was achieved with less effort from the cortex, indicating improved neural efficiency. On the other hand, high-intensity RHG led to higher HbO_2_ levels, which points to inefficient brain activation and possible metabolic strain on cortical circuits. These differential activation patterns reflect an adaptive tuning of neural resources at moderate intensity, enabling more focused and economical processing. This phenomenon aligns with the neural efficiency hypothesis, which asserts that individuals or conditions associated with superior performance exhibit reduced yet more effective neural activation in task-relevant regions (Li and Smith, 2021).

Beyond hemodynamic changes, the cognitive improvements observed under moderate-intensity conditions may also involve neurochemical and neurotrophic mechanisms. Moderate exercise has been shown to elevate levels of brain-derived neurotrophic factor (BDNF), dopamine, and norepinephrine, which enhance synaptic plasticity and frontoparietal network connectivity (Erickson et al., 2011; Chang et al., 2012). These neurochemical modulations may underlie the improved executive control observed in this study, as optimal catecholaminergic activity supports prefrontal efficiency and working memory maintenance. At higher intensities, however, excessive catecholamine release and metabolic stress can disrupt this balance, leading to cognitive fatigue or attentional instability. Thus, moderate-intensity RHG may represent the physiological “sweet spot” where neurovascular coupling, neurotransmitter availability, and cortical efficiency are maximally aligned.

The current findings also enhance our understanding of RHG exercise as a means of cognitive enhancement. While the cognitive benefits of aerobic exercise are well established (Hillman et al., 2008; Stillman et al., 2016), fewer studies have focused on localized resistance exercises. RHG, a simple and accessible form of rhythmic dynamic training, may activate similar neurovascular pathways through increased cardiac output, cerebral blood flow, and cortical oxygenation. Importantly, our study shows that even brief, localized muscle activation can lead to measurable improvements in executive function, especially when performed at moderate intensity. These results support a growing body of evidence suggesting that the magnitude of neural and cognitive benefits depends not only on exercise type and duration, but also on intensity and task specificity. From a theoretical standpoint, these findings indicate a convergence between the inverted-U model of arousal and the neural efficiency hypothesis. Moderate-intensity RHG represents an optimal cortical engagement point where excitation and inhibition are balanced to maximize cognitive output while minimizing neural cost. Such an integrated framework helps explain how exercise intensity modulates cognitive performance and could guide future interventions aimed at improving brain efficiency. Furthermore, these findings provide a neurophysiological basis for incorporating moderate-intensity resistance exercise into cognitive enhancement or rehabilitation programs. From a practical perspective, RHG offers several advantages. It requires minimal equipment, can be performed in limited spaces, and is easily adjustable in intensity, making it feasible for implementation in diverse settings such as workplaces, classrooms, and clinical environments. For individuals at risk of cognitive decline or those with limited mobility, RHG may offer a safe and effective alternative to traditional aerobic programs. The accessibility and adaptability of this exercise modality make it a promising tool for maintaining cognitive vitality across the lifespan.

Despite its strengths, several limitations should be acknowledged. First, the sample consisted exclusively of healthy young male adults, which restricts the generalizability of the findings to other populations, including females, older adults, or individuals with neurological conditions. Second, Systemic and physiological factors may have had some influence on the fNIRS measurements. Although bandpass filtering likely reduced most noise, subtle hemodynamic fluctuations related to cardiac, respiratory, or scalp blood flow could still be present. Incorporating continuous physiological monitoring and advanced regression methods (e.g., short-channel regression or independent component analysis) in future studies can help further enhance the specificity of cortical signal detection during exercise. Third, the lack of a parallel non-exercise control group may have influenced the ability to fully separate exercise effects from potential practice-related improvements. The within-subject, repeated-measures design with randomized sessions likely minimized individual variability and learning bias, though some adaptation effects cannot be completely ruled out. Future research could consider a randomized parallel-group design assigning different participants to low-, moderate-, or high-intensity RHG and a non-exercise control condition to more clearly delineate exercise-specific effects. Finally, the study employed an acute design; therefore, the long-term effects of repeated RHG training on cognition and neural function remain to be explored. Future research should employ longitudinal designs, include a broader demographic range, and incorporate multimodal neuroimaging (e.g., fNIRS–EEG or fNIRS–fMRI) to elucidate the underlying neural mechanisms further.

In conclusion, this study provides robust behavioral and neurophysiological evidence that moderate-intensity RHG exercise enhances executive function by improving prefrontal cortical efficiency. These results highlight the critical role of exercise intensity in shaping cognitive outcomes and suggest that moderate-intensity resistance activity offers an optimal balance between neural activation and metabolic demand. By integrating both behavioral and hemodynamic findings, this study contributes to the growing literature on the neurocognitive effects of exercise. It emphasizes RHG as a practical, low-cost, and effective intervention for enhancing cognitive performance, particularly in young, healthy individuals.

## 5 Conclusion

Our findings show that moderate-intensity RHG exercise at 50% MVC is the most effective for improving cognitive performance in young adults. This level of exercise activates task-related brain regions, enhances neural efficiency, and strengthens executive functions, including inhibitory control and working memory. In contrast, low-intensity exercise at 30% MVC brings limited benefits, while high-intensity exercise at 70% MVC may cause fatigue and reduce cognitive performance.

## Data Availability

The raw data supporting the conclusions of this article will be made available by the authors, without undue reservation.

## References

[ref1] Angevaren M. Vanhees L. Wendel-Vos W. Verhaar H. Aufdemkampe G. Aleman A. . (2007). Intensity, but not duration, of physical activities is related to cognitive function. Eur. J. Cardiovasc. Prev. Rehabil 14, 825–830. doi: 10.1097/HJR.0b013e3282ef995b, PMID: 18043306

[ref2] Arenth P. Ricker J. Schultheis M. (2007). Applications of functional near-infrared spectroscopy (fNIRS) to neurorehabilitation of cognitive disabilities. Clin. Neuropsychol. 21, 38–57. doi: 10.1080/13854040600878785, PMID: 17366277

[ref3] Association (2024). 2024 Alzheimer’s disease facts and figures. (Tech. Rep). Alzheimers Dement. 20, 3708–3821. doi: 10.1002/alz.13809 38689398 PMC11095490

[ref4] Baker L. Bayer-Carter J. Skinner J. Montine T. Cholerton B. Callaghan M. . (2012). High-intensity physical activity modulates diet effects on cerebrospinal amyloid-β levels in normal aging and mild cognitive impairment. J. Alzheimer’s Dis 28, 137–146. doi: 10.3233/JAD-2011-111076, PMID: 21971406 PMC3280092

[ref5] Banerjee S. Smith S. Lamping D. Harwood R. Foley B. Smith P. . (2006). Quality of life in dementia: more than just cognition. An analysis of associations with quality of life in dementia. J. Neurol. Neurosurg. Psychiatry 77, 146–148. doi: 10.1136/jnnp.2005.072983, PMID: 16421113 PMC2077592

[ref6] Batman G. Cooper C. Traylor M. Ransom K. Hill E. Hill B. . (2024). Various modalities of resistance exercise promote similar acute cognitive improvements and hemodynamic increases in young, healthy adults. Cereb. Circ. Cogn. Behav. 7:100363. doi: 10.1016/j.cccb.2024.100363, PMID: 39252851 PMC11381452

[ref7] Bherer L. Erickson K. Liu-Ambrose T. (2013). A review of the effects of physical activity and exercise on cognitive and brain functions in older adults. J. Aging Res. 2013:657508. doi: 10.1155/2013/657508, PMID: 24102028 PMC3786463

[ref8] Brown B. Peiffer J. Sohrabi H. Mondal A. Gupta V. Rainey-Smith S. . (2012). Intense physical activity is associated with cognitive performance in the elderly. Transl. Psychiatry 2:e191. doi: 10.1038/tp.2012.118, PMID: 23168991 PMC3565765

[ref9] Brush C. Olson R. Ehmann P. Osovsky S. Alderman B. (2016). Dose-response and time course effects of acute resistance exercise on executive function. J. Sport Exerc. Psychol. 38, 396–408. doi: 10.1123/jsep.2016-0027, PMID: 27385719

[ref10] Cassilhas R. Viana V. Grassmann V. Santos R. Santos R. Tufik S. . (2007). The impact of resistance exercise on the cognitive function of the elderly. Med. Sci. Sports Exerc. 39, 1401–1407. doi: 10.1249/mss.0b013e318060111f, PMID: 17762374

[ref11] Chang H. Kim K. Jung Y. Kato M. (2017). Effects of acute high-intensity resistance exercise on cognitive function and oxygenation in prefrontal cortex. J. Exerc. Nutr. Biochem. 21, 1–8. doi: 10.20463/jenb.2017.0012, PMID: 28715880 PMC5545209

[ref9001] Chang Y. K. Labban J. D. Gapin J. I. Etnier J. L. (2012). The effects of acute exercise on cognitive performance: a meta-analysis. Brain research. 1453, 87–101. doi: 10.1016/j.brainres.2012.02.068 22480735

[ref12] Colcombe S. Kramer A. (2003). Fitness effects on the cognitive function of older adults: a meta-analytic study. Psychol. Sci. 14, 125–130. doi: 10.1111/1467-9280.t01-1-01430, PMID: 12661673

[ref13] Cummings J. Aisen P. DuBois B. Frölich L. Jack C. J. Jones R. . (2016). Drug development in Alzheimer’s disease: the path to 2025. Alzheimer's Res Ther 8:39. doi: 10.1186/s13195-016-0207-9 27646601 PMC5028936

[ref14] Dallaway N. Lucas S. Marks J. Ring C. (2023). Prior brain endurance training improves endurance exercise performance. Eur. J. Sport Sci. 23, 1269–1278. doi: 10.1080/17461391.2022.2153231, PMID: 36475378

[ref15] de Rooij S. R. (2022). Are brain and cognitive reserve shaped by early life circumstances? Front. Neurosci. 16:825811. doi: 10.3389/fnins.2022.825811, PMID: 35784851 PMC9243389

[ref16] Demurtas J. Schoene D. Torbahn G. Marengoni A. Grande G. Zou L. . (2020). Physical activity and exercise in mild cognitive impairment and dementia: an umbrella review of intervention and observational studies. J. Am. Med. Dir. Assoc. 21, 1415–1422.e6. doi: 10.1016/j.jamda.2020.08.031, PMID: 32981668

[ref17] Dieckelmann M. González-González A. I. Banzer W. Berghold A. Jeitler K. Pantel J. . (2023). Effectiveness of exercise interventions to improve long-term outcomes in people living with mild cognitive impairment: a systematic review and meta-analysis. Sci. Rep. 13:18074. doi: 10.1038/s41598-023-44771-7, PMID: 37872230 PMC10593841

[ref18] Dong M. Li Y. Zhang Y. (2023). The effect of mindfulness training on executive function in youth with depression. Acta Psychol. 235:103888. doi: 10.1016/j.actpsy.2023.103888, PMID: 36934696

[ref19] Erickson K. Voss M. Prakash R. Basak C. Szabo A. Chaddock L. . (2011). Exercise training increases size of hippocampus and improves memory. Proc. Natl. Acad. Sci. USA 108, 3017–3022. doi: 10.1073/pnas.1015950108, PMID: 21282661 PMC3041121

[ref20] Hillman C. Erickson K. Kramer A. (2008). Be smart, exercise your heart: exercise effects on brain and cognition. Nat. Rev. Neurosci. 9, 58–65. doi: 10.1038/nrn2298, PMID: 18094706

[ref21] Hoshi Y. (2003). Functional near-infrared optical imaging: utility and limitations in human brain mapping. Psychophysiology 40, 511–520. doi: 10.1111/1469-8986.00053, PMID: 14570159

[ref22] Huang X. Zhao X. Li B. Cai Y. Zhang S. Wan Q. . (2022). Comparative efficacy of various exercise interventions on cognitive function in patients with mild cognitive impairment or dementia: a systematic review and network meta-analysis. J. Sport Health Sci. 11, 212–223. doi: 10.1016/j.jshs.2021.05.003, PMID: 34004389 PMC9068743

[ref23] Lautenschlager N. Cox K. Flicker L. Foster J. van Bockxmeer F. Xiao J. . (2008). Effect of physical activity on cognitive function in older adults at risk for Alzheimer disease: a randomized trial. JAMA 300, 1027–1037. doi: 10.1001/jama.300.9.1027 18768414

[ref24] Li Q. Feng J. Guo J. Wang Z. Li P. Liu H. . (2013). Effects of the multisensory rehabilitation product for home-based hand training after stroke on cortical activation by using NIRS methods. Neurosci. Lett. 717:134682. doi: 10.1016/j.neulet.2019.134682 31837442

[ref25] Li L. Smith D. (2021). Neural efficiency in athletes: a systematic review. Front. Behav. Neurosci. 15:698555. doi: 10.3389/fnbeh.2021.698555, PMID: 34421553 PMC8374331

[ref26] Liu Y. Lu G. Liu L. He Y. Gong W. (2024). Cognitive reserve over the life course and risk of dementia: a systematic review and meta-analysis. Front. Aging Neurosci. 16:1358992. doi: 10.3389/fnagi.2024.1358992, PMID: 38681665 PMC11047126

[ref27] Liu-Ambrose T. Nagamatsu L. Voss M. Khan K. Handy T. (2012). Resistance training and functional plasticity of the aging brain: a 12-month randomized controlled trial. Neurobiol. Aging 33, 1690–1698. doi: 10.1016/j.neurobiolaging.2011.05.010, PMID: 21741129

[ref28] Mann T. Lamberts R. Lambert M. (2013). Methods of prescribing relative exercise intensity: physiological and practical considerations. Sports Med. 43, 613–625. doi: 10.1007/s40279-013-0045-x, PMID: 23620244

[ref29] Molavi B. Dumont G. A. (2010). Wavelet based motion artifact removal for functional near infrared spectroscopy. Annual International Conference of the IEEE Engineering in Medicine and Biology Society. IEEE Engineering in Medicine and Biology Society. Annual International Conference. 2010, 5–8. 10.1109/IEMBS.2010.5626589 21096093

[ref30] Nelson M. Jester D. Petkus A. Andel R. (2021). Cognitive reserve, Alzheimer’s neuropathology, and risk of dementia: a systematic review and meta-analysis. Neuropsychol. Rev. 31, 233–250. doi: 10.1007/s11065-021-09478-4, PMID: 33415533 PMC7790730

[ref31] Nichols E. Steinmetz J. Vollset S. Fukutaki K. Chalek F. Abd-Allah J. . (2022). Estimation of the global prevalence of dementia in 2019 and forecasted prevalence in 2050: an analysis for the global burden of disease study 2019. Lancet Public Health 7, e105–e125. doi: 10.1016/S2468-2667(21)00249-8 34998485 PMC8810394

[ref32] Noah J. Ono Y. Nomoto Y. Shimada S. Tachibana A. Zhang X. . (2015). fMRI validation of fNIRS measurements during a naturalistic task. J. Vis. Exp. 2015:e52116. doi: 10.3791/52116, PMID: 26132365 PMC4544944

[ref33] Northey J. Cherbuin N. Pumpa K. Smee D. Rattray B. (2018). Exercise interventions for cognitive function in adults older than 50: a systematic review with meta-analysis. Br. J. Sports Med. 52, 154–160. doi: 10.1136/bjsports-2016-096587, PMID: 28438770

[ref34] Parris B. Hasshim N. Wadsley M. Augustinova M. Ferrand L. (2022). The loci of stroop effects: a critical review of methods and evidence for levels of processing contributing to color-word stroop effects and the implications for the loci of attentional selection. Psychol. Res. 86, 1029–1053. doi: 10.1007/s00426-021-01554-x, PMID: 34389901 PMC9090875

[ref35] Pearson A. Miller K. Corkery A. Eisenmann N. Howery A. Cody K. . (2022). Sympathoexcitatory responses to isometric handgrip exercise are associated with white matter hyperintensities in middle-aged and older adults. Front. Aging Neurosci. 14:888470. doi: 10.3389/fnagi.2022.888470, PMID: 35898329 PMC9309556

[ref36] Rorden C. Brett M. (2000). Stereotaxic display of brain lesions. Behav. Neurol. 12, 191–200. doi: 10.1155/2000/421719, PMID: 11568431

[ref9002] Sanchez-Lastra Y. K. Varela S. Cancela J. Ayán C. (2013). Upper versus lower body resistance exercise with elastic bands: effects on cognitive and physical function of institutionalized older adults. European geriatric medicine. 13, 907–916. doi: 10.1007/s41999-022-00616-6 PMC9378322 35150433

[ref37] Sieck D. Ely M. Romero S. Luttrell M. Abdala P. Halliwill J. (2016). Post-exercise syncope: Wingate syncope test and visual-cognitive function. Physiol. Rep. 4:e12883. doi: 10.14814/phy2.12883, PMID: 27550986 PMC5002906

[ref38] Stern Y. (2009). Cognitive reserve. Neuropsychologia 47, 2015–2028. doi: 10.1016/j.neuropsychologia.2009.03.004, PMID: 19467352 PMC2739591

[ref39] Stillman C. Cohen J. Lehman M. Erickson K. (2016). Mediators of physical activity on neurocognitive function: a review at multiple levels of analysis. Front. Neurosci. 10:626. doi: 10.3389/fnhum.2016.00626, PMID: 28018195 PMC5161022

[ref40] Wang Z. Liao M. Li Q. Zhang Y. Liu H. Fan Z. . (2020). Effects of three different rehabilitation games’ interaction on brain activation using functional near-infrared spectroscopy. Physiol. Meas. 41:125005. doi: 10.1088/1361-6579/abcd1f, PMID: 33227728

[ref41] Weuve J. Kang J. Manson J. Breteler M. Ware J. Grodstein F. (2013). Physical activity, including walking, and cognitive function in older women. JAMA 292, 1454–1461. doi: 10.1001/jama.292.12.1454 15383516

[ref42] Zhang S. Zhen K. Su Q. Chen Y. Lv Y. Yu L. (2013). The effect of aerobic exercise on cognitive function in people with Alzheimer’s disease: a systematic review and meta-analysis of randomized controlled trials. Int. J. Environ. Res. Public Health 19:15700. doi: 10.3390/ijerph192315700 PMC9736612 36497772

[ref43] Zhu Y. He S. Herold F. Sun F. Li C. Tao S. . (2022). Effect of isometric handgrip exercise on cognitive function: current evidence, methodology, and safety considerations. Front. Physiol. 13:1012836. doi: 10.3389/fphys.2022.1012836, PMID: 36267588 PMC9576950

[ref44] Zohdi H. Märki J. Scholkmann F. Wolf U. (2024). Cerebral, systemic physiological and behavioral responses to colored light exposure during a cognitive task: a SPA-fNIRS study. Behav. Brain Res. 462:114884. doi: 10.1016/j.bbr.2024.114884, PMID: 38296201

